# Direct but No Transgenerational Effects of Decitabine and Vorinostat on Male Fertility

**DOI:** 10.1371/journal.pone.0117839

**Published:** 2015-02-18

**Authors:** Ruth Kläver, Victoria Sánchez, Oliver S. Damm, Klaus Redmann, Elisabeth Lahrmann, Reinhild Sandhowe-Klaverkamp, Christian Rohde, Joachim Wistuba, Jens Ehmcke, Stefan Schlatt, Jörg Gromoll

**Affiliations:** 1 Institute of Reproductive and Regenerative Biology, Centre of Reproductive Medicine and Andrology, University of Münster, Münster, Germany; 2 Department of Hematology and Oncology, University of Halle, Halle, Germany; 3 Central Animal Facility of the Medical Faculty, University of Münster, Münster, Germany; John A. Burns School of Medicine, UNITED STATES

## Abstract

Establishment and maintenance of the correct epigenetic code is essential for a plethora of physiological pathways and disturbed epigenetic patterns can provoke severe consequences, e.g. tumour formation. In recent years, epigenetic drugs altering the epigenome of tumours actively have been developed for anti-cancer therapies. However, such drugs could potentially also affect other physiological pathways and systems in which intact epigenetic patterns are essential. Amongst those, male fertility is one of the most prominent. Consequently, we addressed possible direct effects of two epigenetic drugs, decitabine and vorinostat, on both, the male germ line and fertility. In addition, we checked for putative transgenerational epigenetic effects on the germ line of subsequent generations (F1–F3). Parental adult male C57Bl/6 mice were treated with either decitabine or vorinostat and analysed as well as three subsequent untreated generations derived from these males. Treatment directly affected several reproductive parameters as testis (decitabine & vorinostat) and epididymis weight, size of accessory sex glands (vorinostat), the height of the seminiferous epithelium and sperm concentration and morphology (decitabine). Furthermore, after decitabine administration, DNA methylation of a number of loci was altered in sperm. However, when analysing fertility of treated mice (fertilisation, litter size and sex ratio), no major effect of the selected epigenetic drugs on male fertility was detected. In subsequent generations (F1–F3 generations) only subtle changes on reproductive organs, sperm parameters and DNA methylation but no overall effect on fertility was observed. Consequently, in mice, decitabine and vorinostat neither affected male fertility *per se* nor caused marked transgenerational effects. We therefore suggest that both drugs do not induce major adverse effects—in terms of male fertility and transgenerational epigenetic inheritance—when used in anti-cancer-therapies.

## Introduction

Epigenetics describes mitotically and/or meiotically stable modifications (such as DNA methylation and histone modification) that function beyond the actual DNA sequence and regulate gene expression [1]. Establishment and maintenance of the correct epigenetic code is essential in physiological pathways involved in development, differentiation and tissue homeostasis. Aberrant epigenetic codes can provoke severe consequences as tumour formation [2,3] and increased DNA methylation as well as decreased histone acetylation seem to play an important role in tumour development. This evidence has lead to the development of epigenetic drugs that aim at inhibition of tumour formation along these epigenetic pathways. So far, two classes of epigenetic drugs are FDA (Food and Drug Administration) approved: DNA methyltransferase (DNMT) and histone deacetylase (HDAC) inhibitors [3–5].

Decitabine, a methyl cytosine analogue (5-aza-2’-deoxycytidine, 5-Aza-CdR), is an example of a DNMT inhibiting drug used in the therapy of myelodysplastic syndrome (MDS) and acute myeloid leukemia (AML). This nucleoside analogue can undergo phosphorylation into a modified pyrimidine, 5-Aza-dCTP (5-aza-2´-deoxycytidine-5´-triphosphate) and is afterwards incorporated irreversibly into nuclear DNA. Following the incorporation of 5-aza-dCTP, DNMTs get covalently bound to DNA and are subsequently degraded, resulting in gradually decreasing DNA methylation during treatment [5,6]. In contrast, the drug vorinostat (suberoylanilide hydroxamic acid, SAHA) belongs to the HDAC (class I and II) inhibitors. It acts by coordinating the zinc ion at the catalytic site provoking an increased histone acetylation. Currently, this drug is used in the therapy of cutaneous T-cell lymphoma (CTCL) [7–9].

In clinical terms, cancer is the prevalent focus of epigenetic research; however, in recent years several studies have also suggested that epigenetics are functionally important for fertility and embryogenesis. Indeed, germ cells exhibit a specific epigenetic code, established in early primordial germ cells (PGCs). These founders of the germ line undergo genome-wide DNA demethylation when entering the gonadal ridge. Independent of gender, this process ensures an equivalent epigenetic state of the germ cells and allows for the subsequent establishment of sex-specific epigenetic germ line modifications. Finally, in males, these major epigenetic changes result in spermatozoa-specific DNA methylation patterns [10–13].

Recent studies have reported associations between aberrant DNA methylation of several imprinted genes in spermatozoa and reduced sperm count, decreased sperm motility and abnormal sperm morphology [14–17]. Furthermore, aberrant methylation patterns of sperm nuclear DNA have been associated with adverse effects on pregnancies and abnormal DNA methylation in the offspring [18–22]. These findings suggest epigenetic processes during spermatogenesis to be specific and highly regulated and that their disruption could have severe consequences for fertilization and subsequent embryogenesis.

Treatment with epigenetic drugs could also induce adverse epigenetic aberrations in germ cells. It might therefore additionally transmit fertility problems and/or severe consequences for the offspring. Previous studies analysing effects of decitabine on the male germ line demonstrated treated mice to present with reduced fertility and impaired semen parameters, i.e. lowered sperm concentration and impaired sperm motility [23,24]. Decitabine treatment of 5-day-old male mice exhibiting spermatogenic differentiation up to premeiotic cells resulted in spermatogenic arrest due to inhibited spermatogonial conversion into spermatocytes [25]. Interestingly, treatment of pregnant mice with decitabine during the period of epigenetic reprogramming (on gestation day 10) induced a reduction of sperm production, pregnancy rate and testis as well as epididymis weight in the F1-generation [26].

These findings point to the possibility that decitabine-induced decline of fertility parameters could also arise in subsequent generations derived from the treated individuals as previous studies described also intergenerational non-genomic effects [27–29]. If intergenerational non-genomic effects result from inherited epigenetic modifications transmitted via the gametes, this phenomenon is referred to as transgenerational epigenetic inheritance [30,31].

As transgenerational epigenetic inheritance is based on a transmission between generations, the analysis of potential transgenerational epigenetic effects implies the investigation of a “new” generation which had not been in direct contact with the stimulus. That means, if a pregnant female is exposed to a factor, the F3-generation has to be studied as F1-generation embryos bearing the F2-generation germ line had already been directly exposed to the stimulus during pregnancy. In case that an individual gets into contact with any exposure postnatally, only the F1-germ line is directly affected and transgenerational epigenetic effects are detectable from the F2-generation onwards [31].

Transgenerational epigenetic effects have been described in a plethora of studies on various issues such as behaviour, late onset diseases or cancer [27–29]. In terms of male fertility, recent studies have elucidated adverse effects on spermatozoal DNA methylation patterns in descendants of treated rodents [32–34]. Nevertheless, despite of numerous reports on this topic, the existence of transgenerational epigenetic inheritance is still under debate as are the included mechanisms. While DNA methylation seems to respond to environmental factors and is mitotically as well as meiotically stable, this epigenetic modification could play a role in the non-genomic transgenerational inheritance [31,35].

Our experiments aimed at the question, whether the treatment of mice with decitabine or vorinostat directly affects male reproductive parameters and fertility and whether transgenerational inherited epigenetic effects on the male germ line and fertility of subsequent generations (F1—F3 generations) exist.

Decitabine and vorinostat were selected based on the fact that these drugs are FDA-approved and already used in anti-cancer therapies. As the number of cancer survivors is currently increasing and some of these patients are even at a reproductive age and might want to father a child after cure and recovery [36,37], it is essential to know whether the used epigenetic drugs could cause direct negative effects on fertility or could induce adverse multi- or transgenerational epigenetic effects in subsequent generations. These experiments are therefore important to gain basic insights on the safety of these treatments on reproduction and offspring.

## Material and Methods

### Study design

We investigated whether the administration of the two epigenetic drugs decitabine and vorinostat has any direct or transgenerational effects on male germ line and fertility. The respective doses were chosen in accordance to the clinical application regimens in patients and with regard to previous studies in mice using these drugs in order to reveal potential risks for patients [23, 24, 38].

Adult male C57Bl/6 mice (7 weeks old, Central Animal Facility of the Medical Faculty, University Münster (original supplier: Charles River, Sulzfeld, Germany)) were chosen for treatment in order to enable sperm analysis. The treatment was performed for seven weeks covering the complete spermatogenetic cycle of approximately 35 days. Thus, every spermatozoon of the treated animals was entirely exposed to the drug during its differentiation [24].

**Fig 1:**
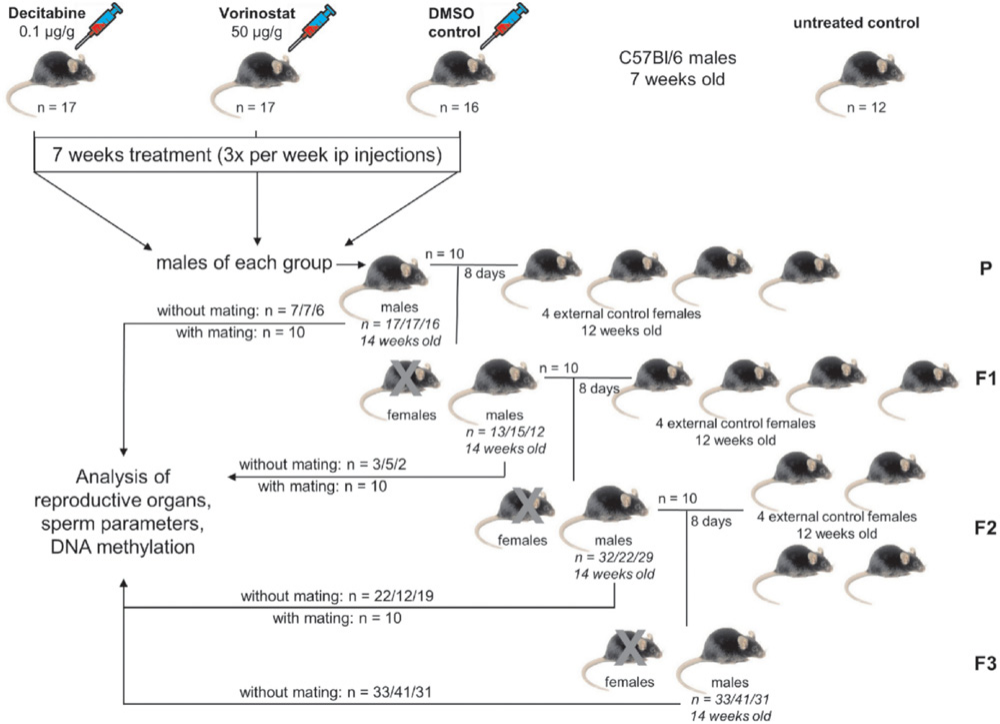
Study design. 62 male C57Bl/6 mice were randomly assigned to four groups receiving either drugs or DMSO or were not treated at all (decitabine, vorinostat, DMSO control, untreated control). Three times per week, mice were injected intraperitoneally for seven weeks. After treatment, 10 males from each treated group were mated simultaneously with four healthy C57Bl/6 females to produce the F1-generation. Identical mating schemes were performed for the F1- and F2-generation. After mating, all male mice of one generation were analysed. Male mice of the F3-generation were not mated but analysed directly after they reached the age of 14 weeks.

To examine direct or transgenerational effects, 10 males per group were mated with four untreated females. The offspring was considered as F1-generation. The F2- and F3-generations were obtained by an identical mating scheme (Fig. 1). Effects detectable in the P-generation were direct effects of the drugs as well as effects observed in the F1-generation. Effects occurring in the F2- or F3-generation were referred to as transgenerational effects. As only males of the P-generation were injected with epigenetic drugs and only male descendants thereof were mated, transmission of drug-related effects is only possible via the male germ line. In order to enable the detection of transgenerational effects, four generations (P, F1, F2 and F3) were analysed.

### Animals

Experimental protocols were approved by the regional/state authority „Landesamt für Natur, Umwelt und Verbraucherschutz Nordrhein-Westfalen” (State Agency for Nature, Environment and Consumer Protection North Rhine-Westphalia) (LANUV AZ 84–02.04.2011.A231) and performed in accordance with the German Federal Law on the care and use of Laboratory animals. 62 male C57Bl/6 mice (P-generation) aged seven weeks were ordered from the Central Animal Facility of the Medical Faculty, University Münster and kept for further 7–8 weeks of the treatment period. All male mice of the F1-generation (n = 30), F2-generation (n = 83) and F3-generation (n = 105) were maintained for 14–15 weeks until mating and subsequent tissue removal. During the study, all mice were housed at 24°C on a 12-h light, 12-h dark cycle and provided with food and tap-water *ad libitum*.

### Treatment

At the age of seven weeks the males of the P-generation were randomly assigned to four groups: decitabine: n = 17, vorinostat: n = 17, vehicle control: n = 16, untreated control: n = 12. Beside the untreated control, all mice were injected intraperitoneally three times per week for seven weeks with either 0.1 mg/kg bodyweight decitabine (5-aza-2′-deoxycytidine, Sigma-Aldrich Corp., St. Louis, MO, USA), 50 mg/kg bodyweight vorinostat (suberoylanilide hydroxamic acid (SAHA), LC Laboratories, Woburn, MA, USA) or with vehicle (7.5% dimethyl sulfoxide (DMSO) in phosphate buffered saline (PBS)). Both drugs were prepared as stock solutions prior to the first injection, divided into several aliquots and stored in tightly sealed vials at -20°C as recommended by the manufacturers. The number of aliquots required was thawed at each injection day at room temperature and injected within 1 hour after thawing.

It is important to note that only the P-generation males were administered with drugs or vehicle, the subsequent F1-, F2- and F3-generations were not subjected to any treatment.

### Mating

From each of the three injected groups 10 randomly selected males were mated simultaneously with four females each for eight days (equals two female cycles) to obtain the F1-generation. At the age of 14 weeks, 10 randomly selected males of the F1-generation per group were mated with four females simultaneously for eight days to obtain the F2-generation and the same mating scheme was performed for the F2-generation to obtain the F3-generation. Males of all generations were mated with 12 week old female C57BL/6 wild type mice (Harlan-Winkelmann, Rossdorf, Germany). Per generation, all mated females were born on the same day to exclude any epigenetic variation due to age.

### Phenotypical observations and organ collections

15 week old (mated and not-mated) male mice were anesthetized by CO_2_ inhalation and killed by decapitation for tissue removal. All animals underwent phenotypical observations and body weights were recorded. Blood samples were collected immediately after decapitation and stored in EDTA-tubes (Sarstedt, Nümbrech, Germany). Testes, epididymides and accessory sex glands (ASG) were weighed before one half of each testis was snap frozen in liquid nitrogen and the other half was fixed in Bouin’s solution over night before it was processed routinely for paraffin embedding for later histological examination.

### Semen analysis

Spermatozoa were isolated from both epididymides (caput, corpus and cauda; as previously described in [39]) by disecting the organs and subsequent incubation in 600 μl Sperm Preparation Medium (Origio, Måløv, Denmark), in which the tissue was minced with fine scissors in order to transfer a maximum of cells into the solution. In order to remove debris, the cell suspension was filtered using a cell strainer (CellTrics, 100 μm, Partec, Münster, Germany). The purity of sperm samples was exemplarily validated by flowcytometrical ploidy analyses (flow cytometer Cytomics FC 500 (Beckman Coulter, Krefeld, Germany), for details see below) of five randomly chosen samples per treatment group of the P-generation (decitabine, vorinostat, vehicle group; only P-generation). The mean percentage of sperm in the samples was 90.24% (± 2.76% standard deviation) and was found to be almost identical in all groups (89.16% (± 3.96%) decitabine; 90.62% (± 2.21%) vorinostat; 90.94% (± 1.95%) vehicle control). Measurements of sperm count, motility, vitality and morphology were carried out according to the WHO guidelines 2010 [40].

### Flow cytometric evaluation of DNA fragmentation with Acridine Orange (FCEAO)

FCEAO was performed as described previously [41]. Briefly, 200 μl TNE buffer including 1 × 10^6^ sperm were mixed with 400 μl of an acid detergent solution (0.08 M HCl, 0.15 M NaCl, 0.1% [vol/vol] Triton X-100, pH 1.2). After exactly 30 seconds, 1.20 ml of acridine orange (AO) staining solution (6 mg AO/ml AO buffer) was added. The AO buffer consisted of 0.037 M citric acid, 0.126 M Na2HPO4, 1.1 mM EDTA disodium, 0.15 M NaCl, pH 6.0. The samples were analysed using the flow cytometer Cytomics FC 500 with an argon laser operated at 488 nm at 40 mW of power. After transiting a 560 nm short-pass dichroic mirror, the green fluorescence was detected through a 525-nm band-pass filter. The red fluorescence was collected through a 675 nm band-pass filter. In total 5000 events were acquired. For the flow cytometer setup and calibration, a “reference” sample was used from a normal mouse semen sample.

Data were analysed with the FCS 3.0 software package (DeNovo software, 3250 Wilshire Blvd. Suite 803, Los Angeles, CA, 90010, USA). A DNA fragmentation index (DFI) for every detected cell was calculated according to the formula: red/(red + green) fluorescence. DFI values were plotted in a histogram.

### Ploidy

Tissue from both testes of all male animals per group were weighed and transferred into a 1.5 ml tube containing 500 μl staining solution (465 μl BSA/PBS (1 mg BSA/ml PBS), 25 μl PI (1 mg propidium iodide /ml PBS), 5 μl RNAse (10 mg RNAse/ml PBS) and 5 μl TritonX-100 (10% Triton X-100 in PBS)). The tissue was minced using scissors, 18 times automatically aspirated by an Eppendorf EDOS 5222 electronic dispensing system (Eppendorf AG, Hamburg, Germany) and two times homogenized for two seconds using an IKA Ultra Turrax tube drive (3.5 speed) (IKA, Staufen, Germany). The solution was filtrated via a cell strainer (CellTrics, 100 μm) to remove residual tissue fragments before it was incubated for 30 minutes in the dark. After incubation, 120 μl of the sample were mixed with 70 μl PBS and 50 μl counting beads and analysed in the flow cytometer Cytomics FC 500 [42]. The cells analysed for DNA content were assigned to categories according to staining intensity: 1) cells with highly condensed DNA (HC: elongated spermatozoa), 2) haploid cells (1C: spermatids), 3) diploid cells (2C: spermatogonia, somatic cells (e.g. Sertoli, peritubular and Leydig cells)), 4) “double diploid” cells (4C: spermatocytes).

### Histological analysis

Six μm sections from Bouin fixed and paraffin embedded tissues were processed and PAS (Periodic acid-Schiff reaction) stained. In order to assess spermatogenic efficiency, a blinded analysis of tubules with spermatozoa within 50 tubules per animal was performed. Additionally, the tubular and luminal diameter and the epithelial height were measured in at least 20 tubules per animal.

### Isolation of DNA and bisulfite conversion

DNA was isolated from blood using FlexiGene DNA Kit (Qiagen, Hilden, Germany) and from 1 × 10^6^ spermatozoa using the Master-Pure DNA Purification Kit (EPICENTRE Biotechnologies, Madison, WI, USA). Afterwards, isolated spermatozoal and blood DNA from up to 20 animals per group were bisulfite converted by the EpiTect Bisulfite Kit (Qiagen, Hilden, Germany).

### Genes of interest

DNA methylation patterns of three maternally methylated (*Mest*, *Lit1* and *Snrpn)* and one paternally methylated imprinted (*H19*) genes were examined as they indicate parent-of-origin expression. Furthermore, Intracisternal-A-Particles (IAPs) were investigated to define the global DNA methylation. One spermatogenesis-specific gene (*Dazl*) and one developmental gene (*Oct4*) were analysed in order to check for potential effects on spermatogenesis and development. As previous studies described an effect of decitabine on *Tcf3* and *Abt1* [24], these transcription factors were also examined.

### Amplification of differentially methylated regions of genes

Differentially methylated regions (DMRs) of the paternally methylated imprinted gene *H19* (chromosome 7; 149766893–149767185, E.v.62), the maternally methylated imprinted genes *Lit1* (GenBank: AJ271885.2), *Snrpn* (chromosome 7; 67149848–67150143, E.v.62), and *Mest* (GenBank: AF017994.1), the spermatogenesis-specific gene *Dazl* (chromosome 17; 50432424–50433024, E.v. 62), the developmental gene *Oct4* (chromosome 17; 35642662–35642991, E.v. 62), the transcription factors *Abt1* (chromosome 13; 23514335–23514735, E.v. 62) and *Tcf3* (chromosome 6; 72577372–72577900, E.v. 62) and the repetitive elements *IAP*s (GenBank: M17551.1) were analysed. PCR primers and the specific thermocycling conditions are listed in the S1 Supporting Information.

### DNA methylation analysis

Quantitative DNA methylation analysis was performed by pyrosequencing (PyroMark Q24 System, Qiagen, Hilden, Germany) on samples from 20 animals per group. For the validation of the assay sensitivity and reproducibility, DNA methylation levels of control samples of different sources (blood, spermatozoa) and of control DNA with different percentages of methylation (Qiagen, Hilden, Germany) were analysed at least in duplicates. Moreover, all pyrosequencing assays included control dispensations at non CpG sites to technically monitor and rule out insufficient bisulfite conversion. In addition, randomly selected samples were analysed in duplicates, in order to prove assay reliability. Pyromark Q24 software (PyroMark Q24 2.0.6.20, Qiagen) was used for analysis. The sequencing primers are listed in the S2 Supporting Information.

### Reduced representation bisulfite sequencing (RRBS)

A total of 100 ng spermatozoal DNA was used for RRBS library preparation using a published protocol with minor modifications already described in detail in previous studies [43,44] (S3 Supporting Information and S1 Table). The RRBS samples were carefully chosen in order to allow optimized analysis of parents and their related F3-generation. We studied representative sperm samples of the vehicle-control (n = 2), decitabine treated animals (n = 3) and their F3-generations (vehicle control: n = 2; decitabine treated animals: n = 3).

### Fertility

For the assessment of the mated males´ fertility the percentage of males which produced offspring, the litter size per male and the sex ratio of the offspring was recorded.

### Statistical calculations

In order to analyse direct effects of decitabine or vorinostat, the treated mice were compared to the DMSO vehicle control, which, in turn, was compared to untreated controls to investigate direct effects of DMSO. In the following generations the decitabine and vorinostat groups were compared to the DMSO vehicle control of the same generation to detect specific transgenerational effects of the drugs. A comparison of the parental DMSO control group with its subsequent generations provided information on non-substance related effects.

All calculations were performed with GraphPad Prism version 5.02 for Windows (GraphPad Software, San Diego, CA, USA). Values were checked for Gaussian distribution by D’Agostino & Pearson omnibus normality test. Statistical differences were detected by unpaired t-test for normally distributed data or by Mann Whitney Test if data did not show normal distribution. Differences were considered to be significant if p < 0.05.

## Results

### Direct effects of decitabine and vorinostat treatment on the P-generation

**Body weight and reproductive tract**

**Fig 2:**
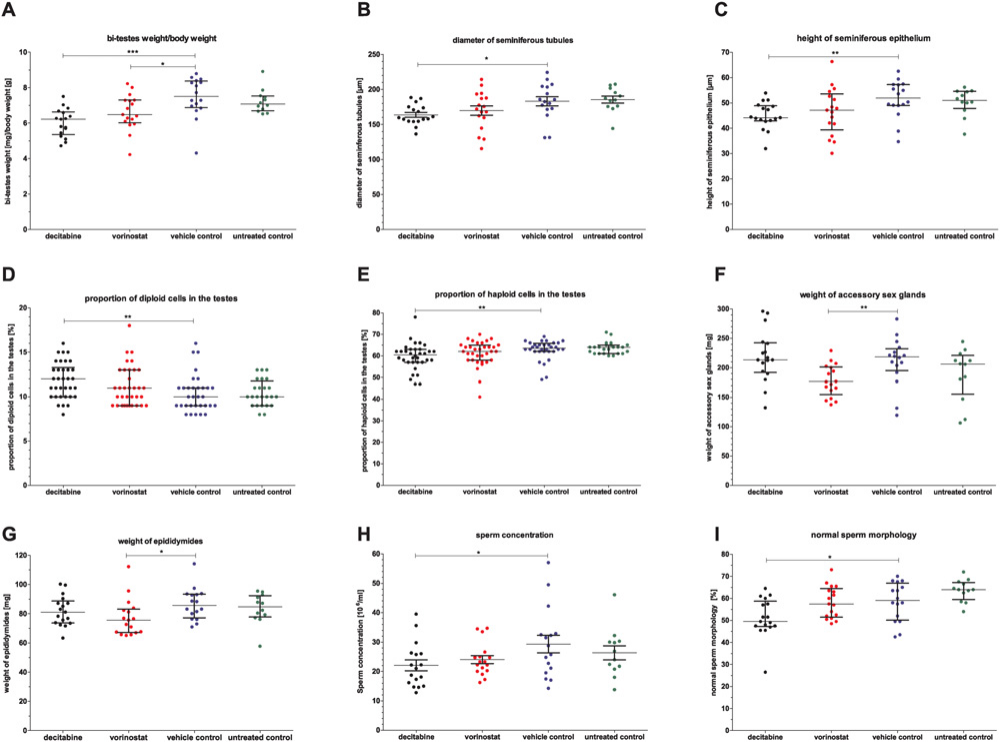
Direct effects of decitabine and vorinostat treatment on reproductive organs and semen parameters. A) Bi-testes weight/body weight, B) diameter of seminiferous tubules, C) height of seminiferous epithelium, D) percentage of diploid cells (undifferentiated germ cells and somatic cells) and E) of haploid cells (spermatids) in the testes, F) weight of accessory sex glands, G) weight of epididymides, H) sperm concentration and I) morphology. One point represents one animal. A), C)—G), I) The median (± interquartile range) or B), H) mean (± SEM) are shown, *: p < 0.05, **: p < 0.01, ***: p < 0.001.

The epigenetic drugs did not affect body weight (S2 Table). However, the treatment with decitabine led to significantly reduced relative testis weight (Fig. 2A), decreased diameter of seminiferous tubules (Fig. 2B) and decreased seminiferous epithelium height (Fig. 2C). Additionally, the proportion of diploid cells was increased (Fig. 2D) and that of haploid testicular cells decreased (Fig. 2E) after decitabine treatment. Treatment with vorinostat affected the reproductive organs, i.e. resulted in decreased relative testis weight (Fig. 2A) as well as smaller accessory sex glands (Fig. 2F) and decreased epididymides weight (Fig. 2G).

**Sperm parameters**

Treatment with decitabine significantly reduced sperm concentration (Fig. 2H) and normal sperm morphology (Fig. 2I), whereas vorinostat did not have any effect (S2 Table).

**DNA methylation**

**Fig 3:**
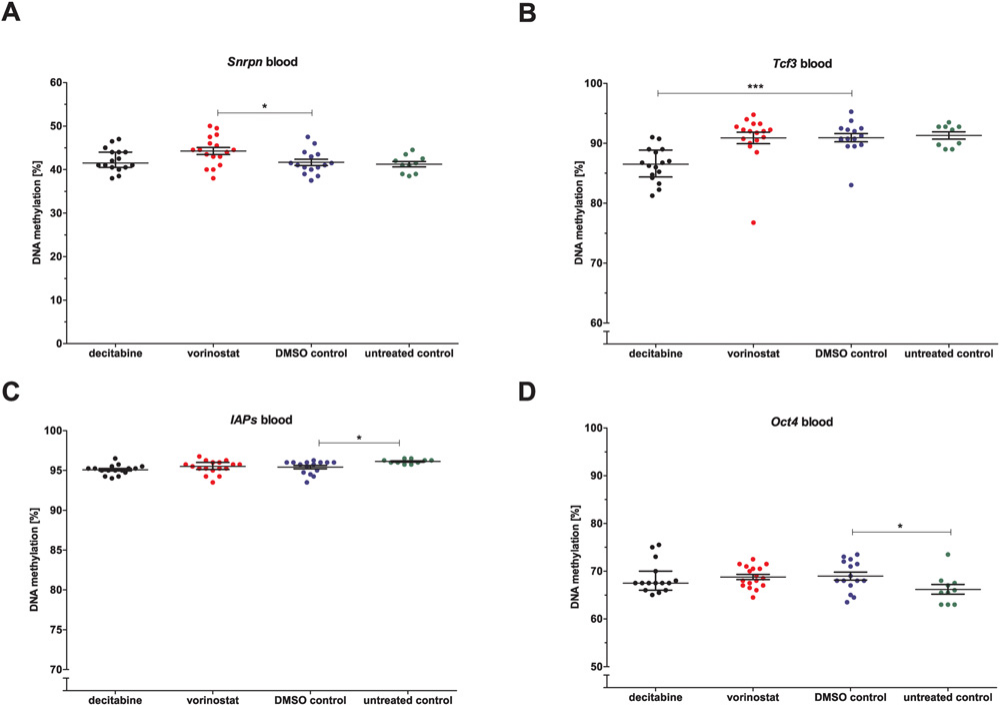
Direct effects of decitabine and vorinostat on DNA methylation in blood of the P-generation. Genes: A) *Snrpn*, B) *Tcf3*, C) *IAPs* and D) *Oct4*. Statistical differences were calculated for decitabine and vorinostat in comparison to DMSO vehicle control and for DMSO vehicle control in comparison to untreated control mice. One point represents one animal. The median (± interquartile range) is shown for each group, *: p < 0.05, **: p < 0.01, ***: p < 0.001, ****: p < 0.0001.

**Fig 4:**
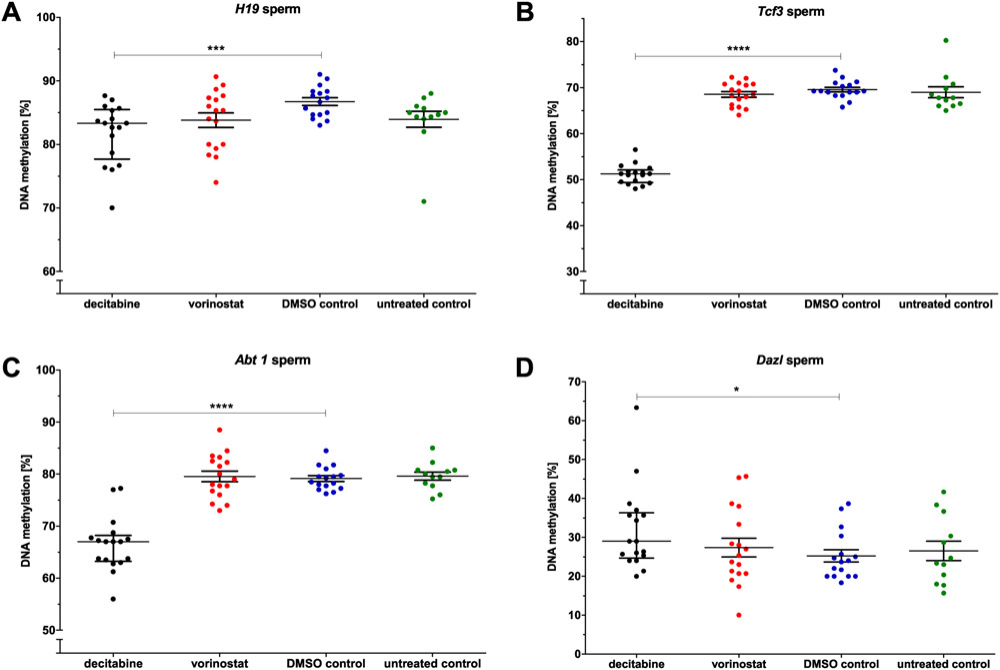
Direct effects of decitabine and vorinostat on DNA methylation in spermatozoa of the P-generation. Genes: A) *H19*, B) *Dazl*, C) *Abt1* and D) *Tcf3*. Statistical differences were calculated for decitabine and vorinostat in comparison to DMSO vehicle control and for DMSO vehicle control in comparison to untreated control mice. One point represents one animal. The median (± interquartile range) is shown for each group, *: p < 0.05, **: p < 0.01, ***: p < 0.001, ****: p < 0.0001.

DNA methylation of some genes in blood and spermatozoa was altered by both epigenetic drugs (S3 Table). Whereas vorinostat led to hypermethylation of *Snrpn* (Fig. 3A), decitabine caused hypomethylation of *Tcf3* (Fig. 3B) in blood. In addition, decitabine treatment resulted in hypomethylation of *H19*, *Tcf3* and *Abt1* in spermatozoa (Fig. 4 A-C). Furthermore, the spermatogenesis-specific gene *Dazl* was hypermethylated in spermatozoa after decitabine treatment (Fig. 4D).

**Fertility**

Whilst litter size and sex ratio of the offspring were not affected by treatment, the number of males producing offspring was slightly reduced after decitabine treatment. Contrary to the vorinostat and DMSO groups in which nine out of 10 mated males produced offspring, in the decitabine group only seven out of 10 mated males reproduced successfully (S1 Fig.).

### Direct effects of decitabine and vorinostat on the F1-generation

**Fig 5:**
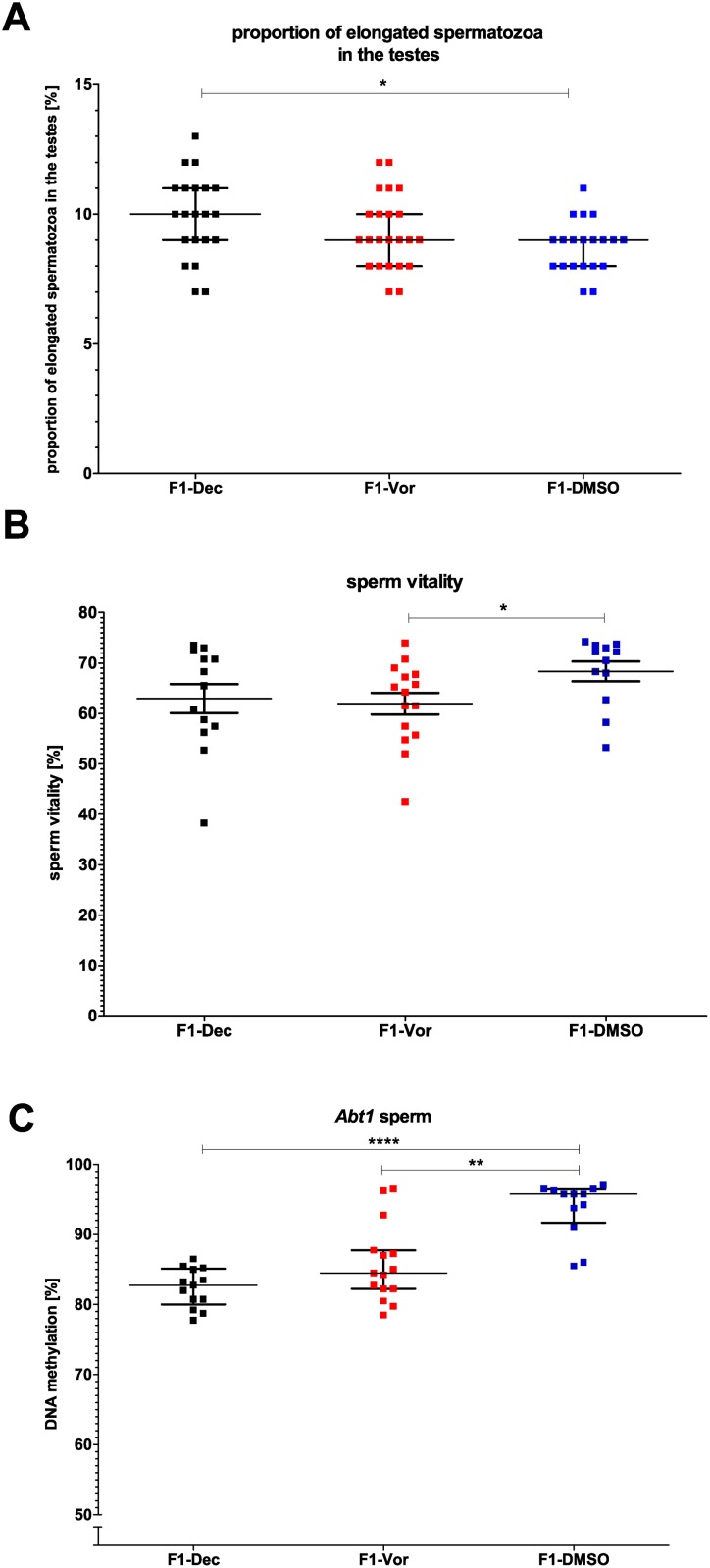
Effects of treatment in the F1-generation. A) Proportion of elongated spermatozoa (cells with highly condensed DNA) in the testes, B) sperm vitality and C) DNA methylation of *Abt1* in spermatozoa. Statistical differences were calculated for decitabine and vorinostat in comparison to DMSO vehicle control. One point represents one animal. A, C) The median (± interquartile range) or B) the mean (± SEM) are shown for each group, *: p < 0.05, **: p < 0.01, ***: p < 0.001, ****: p < 0.0001.

In the F1-generation almost all effects observed in the P-generation had disappeared (S4 Table) and only few remaining changes could be observed: an increased number of elongated spermatids (cells with highly condensed DNA) after decitabine treatment (Fig. 5A) and lower sperm vitality after vorinostat treatment (Fig. 5B). Additionally, *Abt1* was hypomethylated in spermatozoa of the F1-generation derived from both drug-treated groups (Fig. 5C). No effects of decitabine and vorinostat on fertility were found in the F1-generation (S1 Fig.).

### Direct effects of DMSO treatment on the P-generation

Administration of vehicle only (DMSO) did not result in direct effects on body or reproductive organ weights when compared to controls. Similarly, sperm concentration, morphology and DNA fragmentation were not influenced by the vehicle, only progressive sperm motility increased significantly under DMSO treatment (S2 Table). DMSO administration led to a significant DNA hypomethylation of the repetitive elements *IAPs* and a DNA hypermethylation of *Oct4* in blood (Fig. 3C and D). All other DNA methylation patterns of the genes were not affected by DSO treatment, neither in blood nor in spermatozoa (S3 Table).

### Non-substance related effects on F1-, F2- and F3-generations

**Body weight, reproductive tract and sperm parameters**

The comparison of DMSO treated control mice with their subsequent generations revealed some non-substance related effects on body weight (F1, F2 and F3), relative testis weight (F1, F2 and F3), diameter of seminiferous tubules (F2), diameter of seminiferous lumen (F2 and F3), epididymis weight (F2 and F3) and testicular cell composition (F2 and F3). Further unspecific effects could be recognized in the level of DNA fragmentation of nuclear sperm DNA and in sperm motility (F1, F2 and F3), concentration, vitality (F2 and F3) and morphology (F1 and F3) (S5 Table).

**DNA methylation**

Non-substance related altered DNA methylation was found in spermatozoa for all analysed genes in the subsequent generations. Many of these genes displayed aberrant DNA methylation patterns even in all subsequent generations. In blood, less non-substance related effects on DNA methylation patterns were detectable, including the genes *Abt1*, *Tcf3*, *Dazl*, *Lit1* and *IAPs* (S6 Table). However, although significant, most of the differences were only minor.

**Fertility**

Concerning the endpoints recorded for fertility assessment no non-substance related effects were observed (S1 Fig.).

### Transgenerational effects of decitabine and vorinostat on the F2- and F3-generations

**Body weight and reproductive tract**

**Fig 6:**
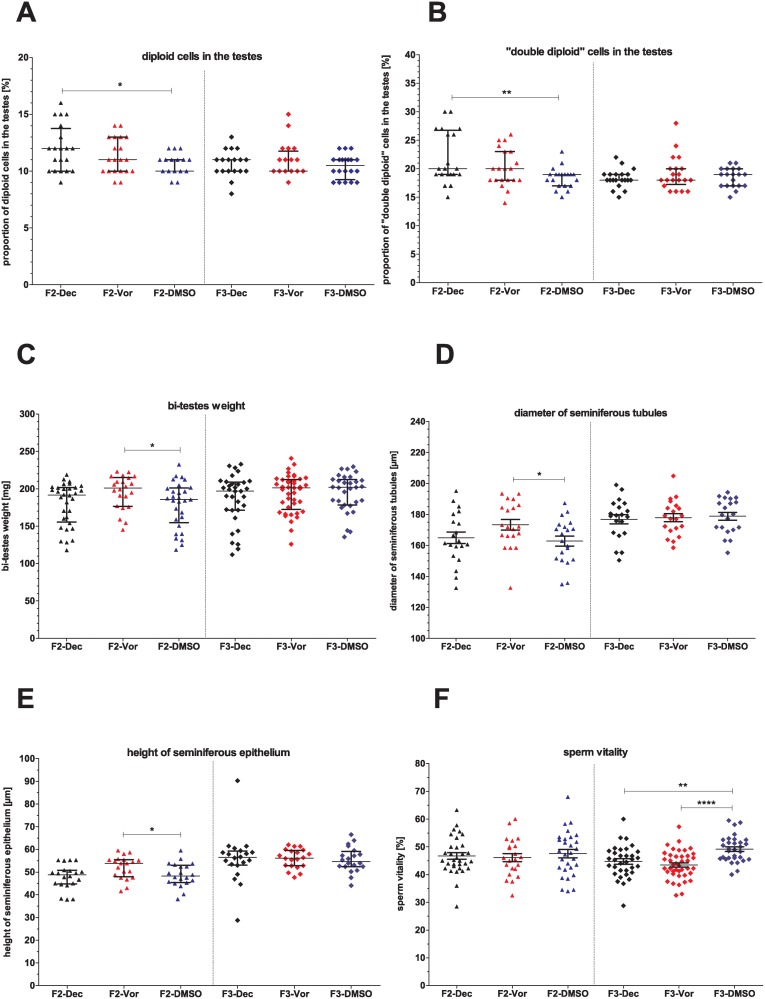
Transgenerational effects of decitabine and vorinostat treatment on reproductive organs and semen parameters. A) Percentage of diploid cells (undifferentiated germ cells and somatic cells) and B) of “double diploid” cells (spermatocytes) in the testes, C) Bi-testes weight, D) diameter of seminiferous tubules, E) height of seminiferous epithelium, F) sperm vitality. One point represents one animal. A)—C), E) The median (± interquartile range) or D), F) the mean (± SEM) are shown, *: p < 0.05, **: p < 0.01, ***: p < 0.001, ****: p < 0.0001.

Decitabine and vorinostat caused only few transgenerational effects (S7 Table). Treatment of mice with decitabine provoked a higher proportion of diploid and “double diploid” cells in the testes of the F2-generation (Fig. 6A and B). Treatment with vorinostat caused an increase of testis weights, mean diameter of seminiferous tubules and height of epithelium in the F2-generation (Fig. 6C—E).

**Sperm parameters**

In the F2- and F3-generations, sperm parameters were almost unaffected by decitabine and vorinostat (S7 Table). Only sperm vitality was decreased in the F3-generation (Fig. 6F).

**DNA methylation**

**Fig 7:**
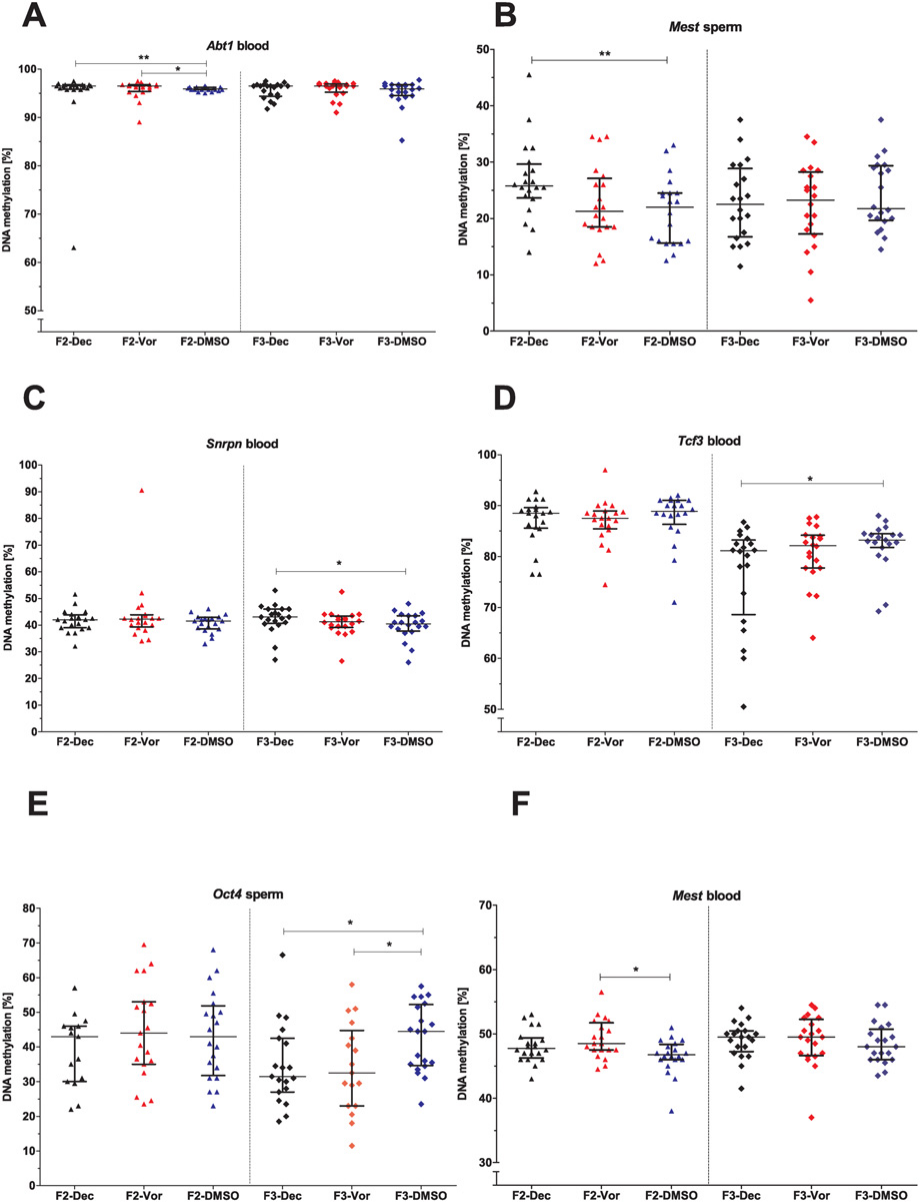
Transgenerational effects of decitabine and vorinostat treatment on DNA methylation levels. Genes: A) *Abt1* in blood, B) *Mest* in spermatozoa, C) *Snrpn* and D) *Tcf3* in blood, E) *Oct4* in spermatozoa, F) *Mest* in blood. One point represents one animal. The median (± interquartile range) is shown for each group, *: p < 0.05, **: p < 0.01.

In blood and spermatozoa DNA methylation patterns of few genes were affected in the F2- and F3-generations subsequently to parental decitabine and vorinostat treatment (S8 Table). Administration of decitabine in the P-generation induced negligible DNA methylation aberrations of *Abt1* in blood (Fig. 7A) and hypermethylation of *Mest* in spermatozoa (Fig. 7B) of the F2-generation. Furthermore, in the F3-generation derived from decitabine-treated animals, hypermethylation of *Snrpn* (Fig. 7C) and hypomethylation of *Tcf3* (Fig. 7D) in blood and hypomethylation of *Oct4* in spermatozoa (Fig. 7E) were detected. In the F2-generation originating from vorinostat-treated animals, hypermethylation of *Mest* (Fig. 7F) and slightly aberrant DNA methylation of *Abt1* (Fig. 7A) in blood were found. Similar to decitabine, vorinostat treatment led to hypomethylation of *Oct4* in spermatozoa of the F3-generation (Fig. 7E).

**Fertility**

Identical with the findings in the F1-generation no differences were found in the proportion of males fathering offspring, litter size and sex ratio of offspring in the F2-generation derived from decitabine- and vorinostat-treated animals compared to the F2-DMSO control group (S1 Fig.).

### Additional analysis of direct and transgenerational effects of decitabine on spermatozoal DNA methylation

**Fig 8:**
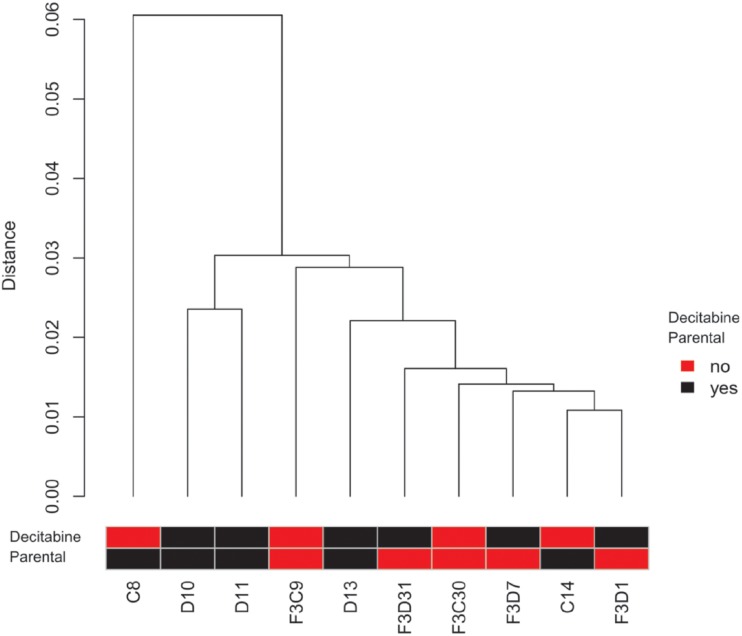
Clustering dendrogram based on DNA methylation of 1.35 × 106 CpG sites (min. coverage 10 reads) present in all samples. Analysed samples: Isolated DNA of spermatozoa of two vehicle-control animals (**C8**, **C14**) and two descendants (analysed sample from the F3-generation of C8: **F3C9** and from the F3-generation of C14: **F3C30**) as well as of three decitabine treated animals (**D10**, **D11**, **D13**) and three descendants (analysed samples from the F3-generation of D10: **F3D1** and **F3D31** and from the F3-generation of D11: **F3D7**). The distance is based on the Pearson’s correlation coefficient that ranges from +1 (two samples are most similar) to-1 (two samples are negatively correlated), while a 0 indicates absence of correlation. To visualize the difference between two samples the distance is calculated by converting the similarity matrix of the Pearson’s correlation coefficient into a distance matrix (1—Pearson’s correlation coefficient) which is then used for hierarchical clustering. Due to the low distance between the samples, our analysis revealed the absence of large differences in the spermatozoal methylome of decitabine treated and untreated animals and their descendants.

Reduced representation bisulfite sequencing (RRBS) was performed to gain information on the genome-wide DNA methylation status after treatment in representative sperm samples of the vehicle-control (n = 2) and decitabine treated animals (n = 3) as well as the respective F3-generations (vehicle control: n = 2; decitabine treated animals: n = 3). In general, the spermatozoal methylomes of the P-generation and F3-generation were highly similar (Fig. 8). However, more detailed analysis of CpG sites revealed some differences between the methylome of decitabine treated and untreated animals (in total 6,821 differentially methylated CpG sites (3,278 hypermethylated and 3,543 hypomethylated)). As about 1/3 of the differentially methylated CpG sites resided in close vicinity to another differentially methylated CpG site, we assume a cooperative signal. Interestingly, these differences disappeared in the F3-generation and only 50 differentially methylated CpG sites remained (Detailed information about RRBS results is given in S4 Supporting Information and S9 Table). Raw data of the RRBS results can be found on the GEO database: http://www.ncbi.nlm.nih.gov/geo/query/acc.cgi?token=mrsdmimgxxebtgx&acc=GSE59575.

## Discussion

Direct and transgenerational effects of the epigenetic drugs decitabine and vorinostat on the male germ line and fertility were addressed in our experiments. Treatment regimens were adapted from clinical use in order to enable the translation of the findings and conclusions into the human situation. The doses chosen mimic those clinically used in cancer patients. However, there is a need for further studies setting up experimental designs that address administration of different doses in order to gain more toxicological information on, an aim which was not in the scope of this study.

Apart from the analysis of the P-generation we obtained three subsequent generations by mating treated males and, subsequently, their untreated descendants to healthy females in order to analyse possible multi- and transgenerational effects.

### Direct effects of decitabine and vorinostat on the P-generation

Drug efficacy was proven by several direct effects observed after administration in the P-generation. Treatment of male mice with decitabine induced a decrease of testicular weight likely caused through the reduced diameter of seminiferous tubules and decreased epithelial height. As the proportion of haploid cells was diminished in the testes and additionally, sperm concentration and normal sperm morphology were impaired, we suggest that decitabine directly interferes with spermatogenic efficiency. This assumption is supported by an increased number of decitabine-induced DNA methylation aberrations in spermatozoa when compared to DNA isolated from blood. Previous studies corroborate our conclusion as they also described a direct effect of decitabine on spermatogenesis. Raman *et al*. (1995) already supposed that decitabine inhibits the differentiation of spermatogonia into spermatocytes as administration of decitabine to 5-days-old prepubertal mice impeded meiotic entry. Thus, they considered DNA methylation as a critical mode of gene regulation for the conversion of spermatogonia into primary spermatocytes [25]. Consistent with this, Trasler and colleagues found that decitabine had an adverse effect on sperm concentration and sperm motility. Additionally, they described a reduction of fertility, i.e. impaired fertilisation ability and a decreased survival of embryos up to the blastocyst stage [23,24]. Although our study confirms similar reproductive effects as observed in these previous studies when applying an identical experimental setting [23,24], we found the effects to be less pronounced. We speculate that such differences might be due to different capacities of the vehicle used to dissolve the substances (saline vs. DMSO), the handling of the drug suspension (fresh vs. frozen) but most likely because of the different genetic background of analysed C57BL/6 colonies.

Furthermore, prenatal treatment seems to have a similar impact on spermatogenesis as treatment of pregnant mice with decitabine on day 10 of gestation adversely affected reproductive parameters in the F1-generation as daily sperm production, pregnancy rate and testicular and epididymal weights [26].

Vorinostat administration directly resulted in smaller testes, epididymides and accessory sex glands; however, in contrast to decitabine, seminiferous tubules, semen parameters and spermatozoal DNA methylation were not altered. Thus, it can be speculated that vorinostat rather affects the reproductive tract more systemically, i.e. that a general influence acts on the endocrine regulation or on somatic components of the reproductive tract or both, an assumption which has to be followed up in future studies. So far, potential effects of vorinostat on the male reproductive system have been only sporadically investigated; i.e. in a single study analysing the effect of vorinostat on male fertility in rats. In this study, no effect on sperm parameters, mating and fertility was detected when doses up to 150 mg/kg/day were given [45]. In contrast, for another HDAC inhibitor (trichostatin-A) it was described that male fertility was hampered by an impairment of spermatogenesis [46].

### Direct effects of decitabine and vorinostat on the F1-generation

Interestingly, most of decitabine- and vorinostat-induced effects described for the P-generation were not observed in the F1-generation. As fertility of the P-generation was not affected by epigenetic drugs, we suggest this lack of effects to be due to selection of normal, healthy and normally methylated spermatozoa during fertilization.

Sperm parameters and DNA methylation measurements reflect the average of the entire sperm population of one individual male but not the individual spermatozoon. That implies, that, although decitabine treatment results in impaired sperm concentration, reduced percentage of normal sperm morphology, and aberrant DNA methylation patterns, there are still some normal, healthy sperm in the ejaculates enabling pregnancy induction. If only those are selected during oocyte fertilization, treatment with epigenetic drugs causing poor sperm parameters and aberrant DNA methylation patterns in a subset of the sperm population may not necessarily have an impact on the health state of the F1-generation.

### Absence of major transgenerational effects of decitabine and vorinostat

The F2- and F3-generations showed no major treatment-induced effects. This absence of gross transgenerational effects is likely due to erasure and renewal of epigenetic marks occurring twice during epigenetic reprogramming: in gametogenesis and after fertilisation in the early zygote. This resetting of the germ cell epigenome avoids the inheritance of acquired somatic epigenetic changes to the offspring and thereby impedes transgenerational epigenetic inheritance [47,48]. However, in contrast to our findings, some studies have already described the transmission of epigenetic effects to the F2-generation after exposure of males of the P-generation [49–51]. In addition, transgenerational effects on the male germ line have been described after treatment *in utero* with various toxic substances. These effects comprised male fertility parameters in the F3-generation, like spermatogenic cell defects and testicular abnormalities. Interestingly, some of these studies identified also sperm epimutations in the F3-generation originated from treated animals [32,33,52]. However, whether this altered sperm epigenome is caused by the same reason as the described somatic effects or induced by a different mechanism is still unclear. It can also not be fully excluded that epigenome alterations of sperm are induced—vice versa—by somatic effects. However, although treatment with different compounds was reported to result in altered gonadal morphology and function of subsequent generations, studies which analysed litter size and sex ratio barely found any impact on fertility of these animals [33,34].

Finally, it is unknown, whether aberrant spermatozoal DNA methylation occurring in treated and subsequent generations has any biological relevance for the offspring. To date no transgenerational study has provided solid evidence for associations of sperm epimutations and fertility (e.g. measured by litter size or pregnancy rate), thus, it appears that such aberrant DNA methylation patterns might not influence reproductive capability.

Nevertheless, genome-wide DNA methylation analysis of higher numbers of samples is needed in future studies to validate whether differentially methylated CpG sites which we detected are persistent also in a larger experimental cohort. As we analysed highly selected samples to optimize the readout, our RRBS results provide first hints to the existence of DNA methylation aberrations that persist in the F3-generation, but only very few of unlikely biological relevance were found.

Undoubtedly, more comprehensive RRBS studies with a higher number of sperm samples are needed as well as the validation of the positive results with an additional assay. Furthermore, additional effects on the subsequent generations should be investigated; for instance, the DNA methylation defects in the P-generation sperm might have an impact on the long term health of the F1 progeny. However, the use of additional assays as well as the analysis of further effects especially in terms of long term health of the progeny were unfeasible due to limitations in sample material in the current study design.

Finally, it should be mentioned that a lower sperm purity (caused by contamination of the sperm sample by somatic cells) can result in altered DNA methylation levels. To exclude this possibility, we measured the sperm purity of randomly chosen parental sperm samples demonstrating a high purity of these samples. As the same isolation procedure was conducted for all four generations it is very unlikely that the sperm purity differs between generations and influences the results of the conducted DNA methylation analyses.

### DMSO and non-substance related effects

Comparing the DMSO control group with untreated mice, a few DMSO related effects were detectable. Additionally, in subsequent generations derived from the DMSO control group, several effects could be observed. These are not necessarily caused by DMSO administration but could also be induced by stress (triggered by injections) [53,54] or other extrinsic factors. Therefore these effects are designated as non-substance-related effects.

Designing the current study, we standardized all factors which putatively could influence mice as much as possible in the experimental setting in order to avoid any non-substance-related effect. However, unknown extrinsic factors could still potentially impact the epigenome. In order to clarify whether effects were due to DMSO or induced by stress due to experimental handling, subsequent generations injected with saline only should be additionally analysed as this question was not in the focus of the presented study. Nevertheless, as some observed effects could be due to DMSO, the use of vehicle should be considered with caution in future experiments.

### Conclusions of the experimental work for the use of decitabine and vorinostat in clinics

Although some direct effects of treatment with decitabine and vorinostat were detectable in the P-generation, neither severe direct nor transgenerational consequences on male fertility were observed. Thus, we can rule out major adverse effects on male fertility occurring due to the administration of these drugs in our experimental setting.

Concluding from these single dose findings in mice we have found little evidence pointing to biologically relevant modifications suggesting that treatment with decitabine and vorinostat may not affect fertility in patients. However, as there are remarkable differences concerning spermatogenesis and spermatogonial stem cell systems between rodents and primates (including the human [55]), definite proof has to be conducted in patients after finishing chemotherapeutic treatment. So far, it may be advisable to suggest waiting at least 1–2 cycles before conceiving (as it is been handled for other anti-cancer agents) to exclude any residual epimutations which might be present in sperm of recently treated men.

## Supporting Information

S1 Fig Fertility parameters. (DOC)

S1 Supporting Information PCR thermocycling conditions and Primers. (DOC)

S2 Supporting Information Sequencing Primers. (DOC)

S3 Supporting Information Material and Methods: Analysis of genome-wide DNA methylation levels by reduced representation bisulfite sequencing. (DOC)

S4 Supporting Information Results: Analysis of genome-wide DNA methylation levels by reduced representation bisulfite sequencing. (DOC)

S1 Table Information about number of total reads and uniquely mapped reads as well as bisulfite conversion efficiency per RRBS sample. (DOC)

S2 Table Body weights, data of reproductive organs and sperm parameters of the treated P-generation. (DOC)

S3 Table DNA methylation of blood and spermatozoa of the treated P-generation. (DOC)

S4 Table Body weights, data of reproductive organs, sperm parameters and DNA methylation of the F1-generation. (DOC)

S5 Table Body weights, data of reproductive organs and sperm parameters of DMSO treated control group and subsequent generations. (DOC)

S6 Table DNA methylation of blood and spermatozoa of the DMSO treated control group and subsequent generations. (DOC)

S7 Table Body weights, data of reproductive organs and sperm parameters of the F2- and F3-generations. (DOC)

S8 Table DNA methylation of blood and spermatozoa of the F2- and F3-generations. (DOC)

S9 Table Differentially methylated CpGs between decitabine treated and control animals from parental generation which persist in F3 generation. (XLS)
