# Supplementary material for: Direct but No Transgenerational Effects of Decitabine and Vorinostat on Male Fertility
Source: PLoS One. 2015 Feb 18;10(2):e0117839. doi: 10.1371/journal.pone.0117839 (PMC4334483; doi:10.1371/journal.pone.0117839)
Supplement: S1 Fig — (DOC) [file pone.0117839.s001.doc]

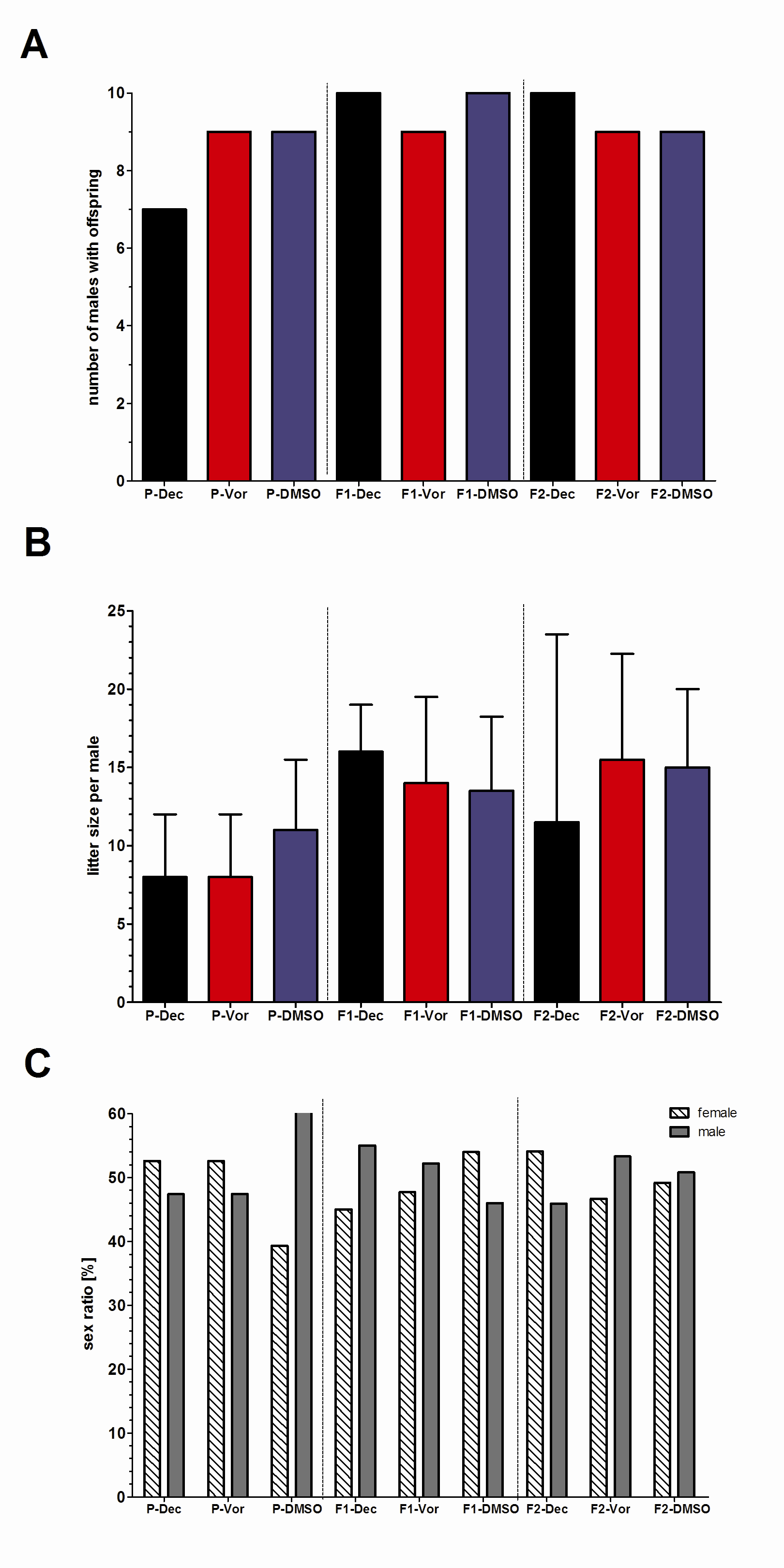


**Figure S1**: **Fertility parameters.** A) Number of males with offspring, B) litter size per male and C) sex ratio. Data are shown as A) absolute number, B) median with interquartile range and C) percentage of all pups per group.
