# Supplementary material for: Direct but No Transgenerational Effects of Decitabine and Vorinostat on Male Fertility
Source: PLoS One. 2015 Feb 18;10(2):e0117839. doi: 10.1371/journal.pone.0117839 (PMC4334483; doi:10.1371/journal.pone.0117839)
Supplement: S1 Supporting Information — (DOC) [file pone.0117839.s002.doc]

**Supporting Information**

***Supporting Information S1***

*PCR thermocycling conditions and Primers*

*a) PCR thermocycling conditions*

PCR mixture (20 µl) for *IAPs*: 1x PCR Reaction Buffer (Roche Applied Sciences, Mannheim, Germany), 200 µM of each dNTP (Roche Applied Sciences, Mannheim, Germany), 2 U FastStart Taq DNA Polymerase (Roche Applied Sciences, Mannheim, Germany), 1 µl of each primer (eurofins mwg, Ebersberg, Germany), 3 µl bisulfite converted DNA. For *Lit1, Mest, Dazl, Abt1, Tcf3, H19, Snrpn* and *Oct4* the reaction mixture comprised: 1x PyroMark PCR Master Mix (Qiagen, Hilden, Germany), 1 µl of each primer (eurofins mwg, Ebersberg, Germany), 3 µl bisulfite converted DNA.

Thermocycling conditions were for *IAPs*: 5 min 95 °C, 35 cycles (30 sec 96 °C; 30 sec 55 °C; 60 sec 72 °C), 7 min 72 °C and 5 min 95 °C, 30 cycles (30 sec 96 °C; 30 sec 61 °C; 30 sec 72 °C), 7 min 72 °C; for *Lit1, Mest, Dazl, Abt1* and *Tcf3*: 15 min 95 °C, 45 cycles (30 sec 95 °C, 30 sec 56 °C, 30 sec 72 °C); 10 min 72 °C; for *H19, Snrpn* and *Oct4*: 15 min 95 °C, 20 cycles (30 sec 95 °C, 30 sec 56 °C, 30 sec 72 °C); 10 min 72 °C and 15 min 95 °C, 35 cycles (30 sec 95 °C, 30 sec 56 °C, 30 sec 72 °C); 10 min 72 °C.

*b) PCR Primers*

*H19*_fwd_out: 5’-AAATTTTAATTTTGGTTGTTTTTGG-3’ (20 pmol/µl)

*H19*_rev_out: 5’-AATCAATTAAAAAAATAATAAAACCC-3’ (20 pmol/µl)

*H19*_fwd_in: 5’-TGGTTGTTTTTGGAATATAATGTT-3’ (20 pmol/µl)

*H19*_rev_in: 5’-biotin -AAAAACAAAACACCTATACCCTTC-3’ (20 pmol/µl)

*LIT1*_fwd: 5´-GTTGGTTATATAGGGTTATAAAGTTTAGGG–3´ (20 pmol/µl)

*LIT1*_rev: 5´-biotin–ATCCCAAAATAAATAACCTAAAACACC-3´ (20 pmol/µl)

*SNRPN*_fwd_out: 5’-TTGGTAGTTGTTTTTTGGTAGGAT-3’ (20 pmol/µl)

*SNRPN*_rev_out: 5’-ATAAACCCAAATCTAAAATATTTTAATCA-3’ (20 pmol/µl)

*SNRPN*_fwd_in: 5’-biotin-TTGGTAGTTGTTTTTTGGTAGGAT-3’ (20 pmol/µl)

*SNRPN*_rev_in: 5’-TAAAATACACTTTCACTACTAAAATCCAC-3’ (20 pmol/µl)

*MEST_*fwd: 5´-AGGTGTTGGTATTTTTAGTGTTAG -3`(20 pmol/µl)

*MEST*_rev: 5´-biotin-CCTCTCTTCCAAACCTAATTTACTACAACT-3`(20 pmol/µl)

*DAZL*_fwd: 5’-GGTAAAATTTAGAAGGTGGAGTAGAAGT-3’ (20 pmol/µl)

*DAZL*_rev: 5´-biotin-AACCAACCACCCACAATCATCC-3’ (20 pmol/µl)

*Oct4*_fwd_out: 5’-TTGAGTGGGTTGTAAGGATAGG-3’ (20 pmol/µl)

*Oct4*_rev_out: 5’- AAAAAATTTCACCTCTCCCTCC-3’ (20 pmol/µl)

*Oct4*_fwd_in: 5’-GTAGGGGTGAGAGGATTTTGAA-3’ (20 pmol/µl)

*Oct4*_rev_in: 5’-biotin-CCACCCTCTAACCTTAACCTCT-3’ (20 pmol/µl)

*IAPs_*fwd_out: 5´-GGT TAG GAA GAA TAT WAT AGA TTA GAA TTT-3`(20 pmol/µl)

*IAPs*_rev_out: 5´-TTC TCT ACT CCA TAT ACT CTA CCT T-3`(20 pmol/µl)

*IAPs_*fwd_in: 5´-GTG TTA TTT TTT GAT TGG TTG TAG TT-3`(20 pmol/µl)

*IAPs*_rev_in: 5´-biotin-TTC TCT ACT CCA TAT ACT CTA CCT-3`(20 pmol/µl)

*Abt1_*fwd: 5´-GGGATGTTTATTGTGTTTGGTTTATAGATT-3`(20 pmol/µl)

*Abt1*_rev: 5´-biotin- CCTTACTATACTTAACTCCCTTCTTTC-3`(20 pmol/µl)

*Tcf3_*fwd: 5´-TGGGGGTTGGTTGTTTATT -3`(20 pmol/µl)

*Tcf3*_rev: 5´-biotin-CCCCCTAAAAAACCTTATAATAACCCTA-3`(20 pmol/µl)
