# Supplementary material for: Direct but No Transgenerational Effects of Decitabine and Vorinostat on Male Fertility
Source: PLoS One. 2015 Feb 18;10(2):e0117839. doi: 10.1371/journal.pone.0117839 (PMC4334483; doi:10.1371/journal.pone.0117839)
Supplement: S2 Supporting Information — (DOC) [file pone.0117839.s003.doc]

***Supporting Information S2***

*Sequencing Primers*

*H19*_seq: 5’-TTTAAGATGATAGTTATTAG-3’ (25 pmol/µl)

*LIT1*_seq: 5´-ATTAGATTAGGTTTTAGTTTTAGTG-3´ (25 pmol/µl)

*MEST*_seq: 5´-ATTTATTGTAGTTTTTTGGTTTAA -3´ (25 pmol/µl)

*SNRPN*_seq: 5’-TCCCAAACAATAACTA-3’ (25 pmol/µl)

*DAZL*_seq: 5’-AAGGTGGAGTAGAAGTTA-3’ (25 pmol/µl)

*Oct4*_seq: 5´-GTTTGGAAGATATAGGTAGA-3´ (25 pmol/µl)

*IAPs*_seq: 5´-ATT TTT TGA TTG GTT GTA GTT TA-3´ (25 pmol/µl)

*Abt1*_seq: 5´-TGTGTTTGGTTTATAGATTATTT-3´ (25 pmol/µl)

*Tcf3*_seq: 5´-GTTTATTTGGTTGGAGTT-3´ (25 pmol/µl)
