# Supplementary material for: Direct but No Transgenerational Effects of Decitabine and Vorinostat on Male Fertility
Source: PLoS One. 2015 Feb 18;10(2):e0117839. doi: 10.1371/journal.pone.0117839 (PMC4334483; doi:10.1371/journal.pone.0117839)
Supplement: S3 Supporting Information — (DOC) [file pone.0117839.s004.doc]

***Supporting Information S3***

*Material and Methods:*

*Analysis of genome-wide DNA methylation levels by reduced representation bisulfite sequencing*

A total of 100 ng DNA was used for reduced representation bisulfite sequencing (RRBS) library preparation using a published protocol with minor modifications (Smith et al, 2009). Briefly, genomic DNA was digested with MspI (NEB, Ipswich, MA, USA), end-repaired and A-tailed with the Klenow-fragment (3'→5' exo-) enzyme (NEB, Ipswich, MA, USA), and ligated using concentrated T4-DNA ligase (NEB, Ipswich, MA, USA) with Illumina TruSeq adapters (Illumina Inc., San Diego, CA). Fragments in a range of 40 to 280 bps insert size were purified from a SYBR gold (Invitrogen) pre-stained agarose gel (NuSieve 3:1 Agarose, Lonza, Allenda, NJ, USA). Libraries were bisulfite converted using the EZ DNA Methylation™ Kit (ZymoResearch, Irvine, CA, USA) and amplified using PfuTurboCx polymerase (Agilent, Santa Clara, CA, USA). The libraries were sequenced on an HiScanSQ instrument (Illumina Inc., San Diego, CA) with version 3 sequencing chemistry. Libraries were spiked during sequencing with 45% PhiX DNA to counteract the imbalance in nucleotide representation. Basecalls were performed using on-instrument real time analysis (RTA) on an Illumina HiScan-SQ. CASAVA version 1.8.2 was used to demultiplex the samples using the inherent TruSeq barcode and export raw sequencing data in fastq format.

Illumina paired-end adapter sequences and 3'-MspI-sites were removed using Trim Galore! version 0.3.3 with implemented Cutadapt version 1.1 The reads were mapped to mouse genome (mm10) which had been retrieved together with RefSeq gene annotation from the University of California Santa Cruz Genome Browser database. Methylation calls from Bismark were extracted with the Bismark methylation_extractor script. We calculated the conversion rate of all non-CpG cytosine positions from the Bismark methylation_extractor output. All samples had a good conversion rate of at least 99.8% of all non-CpG cytosine positions being converted to uracil (Table S1). Sequencing data can be downloaded from NCBI Gene Expression Omnibus (GEO) platform (GSE59575) [Reviewer link: http://www.ncbi.nlm.nih.gov/geo/query/acc.cgi?token=mrsdmimgxxebtgx&acc=GSE59575].

We utilized methylKit to determine differentially methylated cytosines on single CpG resolution. We limited the analysis to CpG sites with a Coverage of at least 10 reads. Within methylKit we combined forward and reverse methylation calls from the same CpG methylation unit using the option destrand=TRUE. Cytosines were identified to be differentially methylated if the methylation difference between two groups was at least 25 percent and the qvalue was at least 0.01.
