# Supplementary material for: Direct but No Transgenerational Effects of Decitabine and Vorinostat on Male Fertility
Source: PLoS One. 2015 Feb 18;10(2):e0117839. doi: 10.1371/journal.pone.0117839 (PMC4334483; doi:10.1371/journal.pone.0117839)
Supplement: S4 Supporting Information — (DOC) [file pone.0117839.s005.doc]

***Supporting Information S4***

*Results:*

*Analysis of genome-wide DNA methylation levels by reduced representation bisulfite sequencing*

We compared the difference in DNA methylation between decitabine treated and vehicle control animals. During the analysis with methylKit 922,295 CpG sites from parental data set and 1,047,534 CpG sites from the F3 data set were included. When we compared decitabine treated mice to vehicle controls, we found a total of 6,821 CpG sites to be differentially methylated. In detail, 3,543 CpG sites were hypomethylated, while 3,278 were found to be hypermethylated in decitabine treated mice.

Next, we investigated if these changes in DNA methylation persist or disappear in the F3 generation. In total 6,261 CpG sites out of 6,821 CpG sites which were found to be differentially methylated in the parental mice, were covered in the F3 comparsion as well. Therefore, we evaluated the difference in DNA methylation between decitabine treated mice and vehicle control mice in the F3 generation at these sites. Surprisingly, we observed that the vast majority was not found to be differentially methylated any more. In detail, 17 sites remained hypomethylated between decitabine treated animals and vehicle controls, while 33 sites remained hypermethylated. Table S9 shows a list of these differentially methylated CpG sites.
