# Supplementary material for: Direct but No Transgenerational Effects of Decitabine and Vorinostat on Male Fertility
Source: PLoS One. 2015 Feb 18;10(2):e0117839. doi: 10.1371/journal.pone.0117839 (PMC4334483; doi:10.1371/journal.pone.0117839)
Supplement: S1 Table — (DOC) [file pone.0117839.s006.doc]

***Table S1: Information about number of total reads and uniquely mapped reads as well as bisulfite conversion efficiency per RRBS sample.***

| **Sample_ID** | **C8** | **C14** | **D10** | **D11** | **D13** | **F3C9** | **F3C30** | **F3D1** | **F3D7** | **F3D31** |
| --- | --- | --- | --- | --- | --- | --- | --- | --- | --- | --- |
| **Total reads** | 28876643 | 40024897 | 32893023 | 30266309 | 26333900 | 42551526 | 37222786 | 53102703 | 32778038 | 32469044 |
| **Uniquely mapped reads** | 15818736 | 23402402 | 19714910 | 18763186 | 15354522 | 25407864 | 21922051 | 33553796 | 19668875 | 19941516 |
| **Conversion efficiency [%]** | 99.87 | 99.90 | 99.88 | 99.87 | 99.90 | 99.88 | 99.88 | 99.92 | 99.82 | 99.87 |
