# Supplementary material for: Direct but No Transgenerational Effects of Decitabine and Vorinostat on Male Fertility
Source: PLoS One. 2015 Feb 18;10(2):e0117839. doi: 10.1371/journal.pone.0117839 (PMC4334483; doi:10.1371/journal.pone.0117839)
Supplement: S2 Table — (DOC) [file pone.0117839.s007.doc]

**Table S2**: **Body weights, data of reproductive organs and sperm parameters of the treated P-generation.** Data are shown as mean (± SEM) and median (with range). Statistical differences were calculated for decitabine and vorinostat in comparison to DMSO vehicle control and for DMSO vehicle control in comparison to untreated control group (shown as p-value). Significant direct effects of decitabine, vorinostat and DMSO treatment are marked in grey.

| **P-generation** | **decitabine**  **(n = 17)** | **vorinostat**  **(n = 17)** | **DMSO control**  **(n = 16)** | **untreated control**  **(n = 12)** |
| --- | --- | --- | --- | --- |
| **Body weight [g]** | 24.94 (± 0.41) 25 (22 - 29) p = 0.80 | 24.76 (± 0.44) 25 (21 - 27) p = 0.99 | 24.88 (± 0.35) 24.5 (23 - 29) p = 0.09 | 26 (± 0.54) 26 (23 – 29) |
| **Testes weight [mg]** | 149.9 (± 4.37) 156.2 (122.5 - 172.4) p = 0.0001 | 163.5 (± 6.12) 170.8 (114 - 196.1) p = 0.017 | 184.4 (± 5.94) 183.6 (125 - 211) p = 0.95 | 187.3 (± 4.13) 186.2 (164.5 - 213.9) |
| **Testes weight/body weight [mg/g]** | 6.027 (± 0.19) 6.22 (4.72 - 7.5) p = 0.0003 | 6.62 (± 0.24) 6.48 (4.22 - 8.22) p = 0.014 | 7.46 (± 0.29) 7.505 (4.31 - 8.79) p = 0.31 | 7.23 (± 0.19) 7.075 (6.52 - 8.91) |
| **ASG weight [mg]** | 219 (± 10.68) 213.2 (132.2 - 296.3) p = 0.82 | 177.9 (± 6.55) 176.3 (137.3 - 229) p = 0.006 | 210.2 (± 10.35) 218.3 (119.4 - 283) p = 0.20 | 188.7 (± 12.98) 206 (106.2 – 244.3) |
| **Epididymides weight [mg]** | 81.93 (± 2.49) 81.1 (63.3 - 100.3) p = 0.26 | 77.3 (± 3.05) 75.6 (65.2 - 112.2) p = 0.014 | 86.61 (± 2.74) 85.75 (71 - 114.1) p = 0.69 | 83.55 (± 3.05) 84.75 (57.8 – 95.5) |
| **Diameter of sem.tubules [µm]** | 163.7 (± 3.47) 159.7 (136.3 - 188.4) p = 0.013 | 169.7 (± 6.67) 172 (115.5 - 214.3) p = 0.17 | 183 (± 6.56) 183.9 (131 - 224.4) p = 0.79 | 185.4 (± 5.04) 186 (144.1 - 207.3) |
| **Height of sem.epithelium [µm]** | 44.94 (± 1.29) 44.14 (31.94 - 53.86) p = 0.004 | 46.58 (± 2.27) 47.1 (30.04 - 66.34) p = 0.06 | 51.49 (± 1.85) 51.93 (34.7 - 62.52) p = 0.50 | 50.3 (± 1.54) 50.98 (37.65 - 56.2) |
| **Diameter of sem.lumen [µm]** | 73.87 (± 2.18) 73.05 (56.84 - 92.36) p = 0.31 | 76.54 (± 2.75) 78.7 (55.19 - 97.64) p = 0.53 | 80.02 (± 3.70) 82.95 (53.81 - 109.9) p = 0.42 | 84.8 (± 2.47) 84.1 (68.77 - 98.02) |
| **Composition of testes: HC [%]** | 8.059 (± 0.38) 8 (3 - 16) p = 0.75 | 8.118 (± 0.44) 8 (3 - 14) p = 0.75 | 7.84 (± 0.39) 7.5 (4 - 13) p = 0.48 | 8.29 (± 0.44) 8 (5 - 12) |
| **Composition of testes: 1C [%]** | 59.62 (± 1.06) 60.5 (47 - 78) p = 0.002 | 61.21 (± 0.99) 62 (41 - 70) p = 0.18 | 62.78 (± 0.79) 63.5 (49 - 69) p = 0.76 | 63.63 (± 0.57) 64 (60 - 71) |
| **Composition of testes: 2C [%]** | 11.85 (± 0.35) 12 (8 - 16) p = 0.003 | 11.18 (± 0.38) 11 (9 - 18) p = 0.12 | 10.38 (± 0.37) 10 (8 - 16) p = 0.56 | 10.42 (± 0.31) 10 (8 - 13) |
| **Composition of testes: 4C [%]** | 20.41 (± 0.72) 20 (7 - 30) p = 0.08 | 19.56 (± 0.7) 19 (11 - 32) p = 0.71 | 19.09 (± 0.55) 19 (14 - 28) p = 0.12 | 17.79 (± 0.53) 18 (14 - 23) |
| **Efficiency of spermatogenesis [%]** | 0.97 (± 0.01) 0.98 (0.92 - 1) p = 0.59 | 0.98 (± 0.005) 0.98 (0.94 - 1) p = 0.19 | 0.96 (± 0.006) 0.96 (0.92 - 1) p = 0.54 | 0.97 (± 0.007) 0.97 (0.92 - 1) |
| **Sperm concentration [mill/ml]** | 22.1 (± 1.84) 21 (12.75 - 39.5) p = 0.045 | 24.04 (± 1.36) 23.25 (16.25 - 34.7) p = 0.11 | 29.33 (± 2.99) 27.25 (14.25 - 57) p = 0.47 | 26.33 (± 2.41) 25.45 (13.8 – 46.1) |
| **Progressive sperm motility [%]** | 50.24 (± 2.02) 52.25 (29.5 - 60) p = 0.48 | 55.54 (± 1.51) 55.5 (46.5 - 65.25) p = 0.20 | 52.72 (± 1.3) 54 (44.5 - 60.75) p = 0.024 | 45.85 (± 2.23) 48.13 (33 - 55.75) |
| **Sperm vitality [%]** | 69.18 (± 1.55) 70.5 (55.25 - 79.25) p = 0.56 | 68.75 (± 1.67) 69 (58 - 78.75) p = 0.46 | 70.48 (± 1.58) 71.13 (58.25 - 78) p = 0.38 | 68.29 (± 1.89) 67.25 (59 - 78.5) |
| **Normal sperm morphology [%]** | 51.21 (± 2.16) 49.5 (26.5 - 64.5) p = 0.024 | 58.32 (± 1.76) 57.5 (48.5 - 73) p = 0.75 | 58.5 (± 2.21) 59 (42.5 - 70) p = 0.15 | 63.63 (± 1.43) 64 (54 - 72) |
| **DNA fragmentation [%]** | 3.44 (± 0.74) 2.58 (1.13 - 12.72) p = 0.41 | 2.74 (± 0.35) 2.42 (1.6 - 7.82) p = 0.71 | 3.22 (± 0.96) 2.3 (1.06 - 17.48) p = 0.26 | 2.87 (± 0.85) 1.97 (1.41 – 12.1) |
