# Supplementary material for: Direct but No Transgenerational Effects of Decitabine and Vorinostat on Male Fertility
Source: PLoS One. 2015 Feb 18;10(2):e0117839. doi: 10.1371/journal.pone.0117839 (PMC4334483; doi:10.1371/journal.pone.0117839)
Supplement: S3 Table — (DOC) [file pone.0117839.s008.doc]

**Table S3: DNA methylation of blood and spermatozoa of the treated P-generation.** Data are shown as mean [%] (± SEM) and median [%] (with range). Statistical differences were calculated for decitabine and vorinostat in comparison to DMSO vehicle control and for DMSO vehicle control in comparison to untreated control group (shown as p-value and marked in grey).

| **P-generation** | **decitabine**  **(n = 17)** | **vorinostat**  **(n = 17)** | **DMSO control**  **(n = 16)** | **untreated control**  **(n = 12)** |
| --- | --- | --- | --- | --- |
| **Blood** |  |  |  |  |
| ***IAPs*** | 95.09 (± 0.15) 95.13 (94 - 96.5) p = 0.104 | 95.41 (± 0.20) 95.5 (93.5 - 96.75) p = 0.80 | 95.42 (± 0.21) 95.75 (93.5 - 96.25) p = 0.0103 | 96.13 (± 0.085) 96.13 (95.75 - 96.5) |
| ***Mest*** | 48.78 (± 0.84) 48.5 (43.5 - 55.5) p = 0.16 | 47.82 (± 0.57) 48 (44 - 52) p = 0.45 | 47.07 (± 0.51) 47 (44 - 50.5) p = 0.54 | 48.15 (± 1.01) 47.75 (43.5 - 53.5) |
| ***Lit1*** | 59.35 (± 0.91) 58.67 (53.67 - 69) p = 0.17 | 57.94 (± 0.75) 57.67 (50.33 - 65) p = 0.57 | 57.24 (± 1.07) 57.67 (50 - 67.33) p = 0.51 | 58.17 (± 1.18) 56.83 (55.67 - 68) |
| ***Snrpn*** | 42.22 (± 0.66) 41.5 (38 - 47) p = 0.55 | 44.26 (± 0.81) 44.5 (38 - 50) p = 0.03 | 41.67 (± 0.71) 41 (37.5 - 47.5) p = 0.98 | 41.25 (± 0.63) 41.25 (38.5 - 44.5) |
| ***H19*** | 58.13 (± 1.46) 59 (42.67 - 66) p = 0.57 | 59.24 (± 1.78) 60 (41.33 - 71) p = 0.94 | 59.98 (± 1.20) 59 (52.33 - 68.33) p = 0.74 | 60.3 (± 2.23) 63.83 (46.67 - 67.33) |
| ***Dazl*** | 95.04 (± 0.23) 95 (93.33 - 96.67) p = 0.84 | 95.22 (± 0.20) 95.33 (93.67 - 96.67) p = 0.35 | 95.02 (± 0.20) 95.33 (93.67 - 96.33) p = 0.13 | 95.43 (± 0.12) 95.5 (94.67 - 96) |
| ***Oct4*** | 68.6 (± 0.86) 67.5 (65 - 75.5) p = 0.45 | 68.79 (± 0.55) 68.5 (64.5 - 72.5) p = 0.71 | 68.97 (± 0.82) 68 (63.5 - 73.5) p = 0.04 | 66.2 (± 1) 65.75 (63 - 73.5) |
| ***Abt1*** | 97 (± 0.07) 97.13 (96.5 - 97.25) p = 0.14 | 96.91 (± 0.21) 97 (93.75 - 97.75) p = 0.23 | 97.05 (± 0.21) 97.25 (94.75 - 97.75) p = 0.38 | 97.1 (± 0.09) 97 (96.75 - 97.5) |
| ***Tcf3*** | 86.38 (± 0.71) 86.5 (81.25 - 91) p = 0.0002 | 90.9 (± 0.97) 92 (76.75 - 94.75) p = 0.6 | 90.93 (± 0.70) 90.75 (83 - 95.25) p = 0.81 | 91.31 (± 0.61) 92.25 (89 - 93.5) |
| **Sperm** |  |  |  |  |
| ***IAPs*** | 88.1 (± 0.31) 88.25 (86 - 90.75) p = 0.188 | 87.84 (± 0.55) 87.5 (83.25 - 91.75) p = 0.59 | 87.48 (± 0.34) 87.5 (86 - 91) p = 0.37 | 88 (± 0.47) 87.63 (86 - 90.5) |
| ***Mest*** | 17.32 (± 1.2) 17 (7.5 - 28) p = 0.104 | 16 (± 1.69) 13.5 (7 - 31.5) p = 0.94 | 14.97 (± 1.05) 14.25 (10.5 - 25) p = 0.55 | 16.54 (± 1.6) 15.25 (11 - 29) |
| ***Lit1*** | 23.98 (± 1.6) 22.67 (13.33 - 38.33) p = 0.35 | 23.67 (± 2.24) 22 (8.333 - 44) p = 0.73 | 21.94 (± 1.6) 20.17 (13.33 - 36.67) p = 0.87 | 22.14 (± 2.06) 20.5 (13.67 - 34) |
| ***Snrpn*** | 14.68 (± 1.08) 13.5 (8.5 - 25) p = 0.05 | 14.35 (± 1.60) 12.5 (6 - 31) p = 0.34 | 11.91 (± 0.93) 10.75 (7 - 19.5) p = 0.55 | 12.92 (± 1.2) 12 (8 - 21) |
| ***H19*** | 81.78 (± 1.15) 83.33 (70 - 87.67) p = 0.0009 | 83.8 (± 1.15) 85.33 (74 - 90.67) p = 0.11 | 86.73 (± 0.61) 87 (83 - 91) p = 0.07 | 83.94 (± 1.26) 84.83 (71 - 88) |
| ***Dazl*** | 31.9 (± 2.62) 29 (20 - 63.33) p = 0.024 | 27.37 (± 2.40) 25.33 (10 - 45.67) p = 0.53 | 25.25 (± 1.57) 23.83 (18.33 - 38.67) p = 0.94 | 26.53 (± 2.5) 24 (15.67 - 41.67) |
| ***Oct4*** | 31.06 (± 2.63) 29 (13.5 - 56.5) p = 0.63 | 36.29 (± 3.22) 31.5 (16.5 - 63) p = 0.44 | 31.88 (± 1.85) 30.75 (19 - 49) p = 0.95 | 32.79 (± 3.33) 29.25 (21.5 - 58) |
| ***Abt1*** | 66.54 (± 1.27) 67 (56 - 77.25) p < 0.0001 | 79.56 (± 1.02) 79 (73 - 88.5) p = 0.87 | 79.16 (± 0.56) 78.88 (76.25 - 84.5) p = 0.53 | 79.63 (± 0.76) 79.75 (75.25 - 85) |
| ***Tcf3*** | 51.12 (± 0.51) 51.25 (48 - 56.5) p < 0.0001 | 68.53 (± 0.61) 68.5 (64 - 72.25) p = 0.31 | 69.55 (± 0.49) 69.25 (65.75 - 73.75) p = 0.16 | 68.98 (± 1.19) 67.75 (65 - 80.25) |
