# Supplementary material for: Direct but No Transgenerational Effects of Decitabine and Vorinostat on Male Fertility
Source: PLoS One. 2015 Feb 18;10(2):e0117839. doi: 10.1371/journal.pone.0117839 (PMC4334483; doi:10.1371/journal.pone.0117839)
Supplement: S4 Table — (DOC) [file pone.0117839.s009.doc]

**Table S4**: **Body weights, data of reproductive organs, sperm parameters and DNA methylation of the F1-generation.** Data are shown as mean (± SEM) and median (with range). Statistical differences were calculated for decitabine and vorinostat in comparison to DMSO vehicle control (shown as p-value). Significant direct effects of the decitabine and vorinostat group are marked in grey.

|  | **F1** | | |
| --- | --- | --- | --- |
|  | **Decitabine**  **(n = 13)** | **Vorinostat**  **(n = 15)** | **DMSO-control**  **(n = 12)** |
| **Body weight [g]** | 29.23 (± 0.63) 29 (25 - 32) p = 0.89 | 30.4 (± 0.48) 30 (26 - 34) p = 0.08 | 29.25 (± 0.77) 29 (26 - 35) |
| **Testes weight [mg]** | 186.2 (± 6.49) 195.3 (142.7 - 217.2) p = 0.72 | 194.9 (± 6.82) 204.8 (127.2 - 221.3) p = 0.08 | 181.2 (± 9.09) 198.3 (123.2 - 208) |
| **Testes weight/body weight [mg/g]** | 6.43 (± 0.30) 6.73 (4.6 - 8.18) p = 0.5 | 6.44 (± 0.25) 6.71 (4.1 - 7.88) p = 0.61 | 6.15 (± 0.30) 6.33 (4.56 - 7.69) |
| **ASG weight [mg]** | 187.8 (± 11.71) 182.8 (108 - 256.5) p = 0.61 | 201.7 (± 15.87) 202.2 (36.1 - 278.8) p = 0.58 | 189.5 (± 18.5) 193.8 (51.9 - 270.4) |
| **Epididymides weight [mg]** | 92.62 (± 3.20) 92.5 (71.5 - 114.7) p = 0.72 | 96.21 (± 1.99) 96.3 (83.4 - 108.6) p = 0.49 | 94.96 (± 4.99) 92.6 (67.6 - 127.5) |
| **Diameter of sem.tubules [µm]** | 171.8 (± 2.8) 171.4 (159.7 - 186.2) p = 0.65 | 175.8 (± 3.78) 179.7 (148.2 - 197.2) p = 0.76 | 174.1 (± 4.21) 177 (146.8 - 192.9) |
| **Height of sem.epithelium [µm]** | 48.96 (± 1.68) 47.66 (40.45 - 57.59) p = 0.85 | 49.11 (± 1.55) 51.56 (33.71 - 57.26) p = 0.79 | 48.96 (± 1.59) 47.9 (40.13 - 58.32) |
| **Diameter of sem.lumen [µm]** | 73.85 (± 1.99) 74.89 (59.71 - 85.36) p = 0.77 | 77.64 (± 2.17) 78.33 (62.66 - 92.69) p = 0.48 | 76.17 (± 2.57) 74.75 (65.99 - 97.83) |
| **Composition of testes: HC [%]** | 9.905 (± 0.35) 10 (7 - 13) p = 0.012 | 9.304 (± 0.30) 9 (7 - 12) p = 0.22 | 8.75 (± 0.23) 9 (7 - 11) |
| **Composition of testes: 1C [%]** | 57.14 (± 0.74) 58 (50 - 63) p = 0.71 | 57.78 (± 0.84) 59 (41 - 61) p = 0.61 | 56 (± 1.29) 59 (45 - 61) |
| **Composition of testes: 2C [%]** | 11.05 (± 0.43) 11 (9 - 15) p = 0.06 | 11.13 (± 0.33) 11 (10 - 17) p = 0.15 | 12 (± 0.48) 11 (9 - 16) |
| **Composition of testes: 4C [%]** | 22.14 (± 0.52) 21 (20 - 28) p = 0.61 | 21.78 (± 0.60) 21 (18 - 33) p = 0.29 | 23 (± 0.85) 22 (18 - 31) |
| **Efficiency of spermatogenesis [%]** | 0.98 (± 0.006) 0.98 (0.92 - 1) p = 0.64 | 0.98 (± 0.005) 0.98 (0.94 - 1) p = 0.71 | 0.975 (± 0.01) 0.98 (0.94 - 1) |
| **Sperm concentration [mill/ml]** | 31.85 (± 2.12) 29.67 (19.51 - 47.1) p = 0.49 | 35.85 (± 3.48) 32.99 (19.09 - 64.33) p = 0.79 | 34.52 (± 3.23) 36.98 (13.36 - 47.31) |
| **Progressive sperm motility [%]** | 60.55 (± 2.04) 61.66 (49.75 - 72.25) p = 0.37 | 60.82 (± 1.67) 62.75 (47 - 69) p = 0.66 | 62.73 (± 1.59) 62.13 (52.75 - 71) |
| **Sperm vitality [%]** | 62.96 (± 2.85) 65.5 (38.25 - 73.5) p = 0.14 | 61.97 (± 2.13) 64.25 (42.5 - 74) p = 0.04 | 68.33 (± 1.96) 71.38 (53.25 - 74.25) |
| **Normal sperm morphology [%]** | 63.69 (± 2.72) 66.5 (46.5 - 77.5) p = 0.79 | 60.67 (± 2.55) 60.5 (33.5 - 73) p = 0.17 | 65.42 (± 2.04) 65.75 (53 - 77) |
| **DNA fragmentation [%]** | 6.76 (± 0.79) 6.02 (3.88 - 13.97) p = 0.81 | 8.45 (± 1.85) 5.72 (3.55 - 32.32) p = 0.90 | 7.8 (± 1.27) 6.67 (2.83 - 14.46) |
| **DNA methylation [%]** |  |  |  |
| **Blood** |  |  |  |
| ***IAPs*** | 94.92 (± 0.082) 95 (94.5 - 95.25) p = 0.59 | 95.05 (± 0.13) 95.25 (93.5 - 95.75) p = 0.49 | 95.02 (± 0.13) 95 (94.25 - 96) |
| ***Mest*** | 46.62 (± 2.1) 45.5 (35.5 - 67) p = 0.94 | 48.4 (± 1.49) 47.5 (40 - 61.5) p = 0.07 | 45.33 (± 0.70) 45.75 (41 - 49) |
| ***Lit1*** | 60.9 (± 1.47) 59.67 (51.67 - 70.67) p = 0.06 | 61.13 (± 1.56) 60.33 (51 - 72.67) p = 0.06 | 56.94 (± 1.14) 58.17 (47.67 - 62.67) |
| ***Snrpn*** | 42.46 (± 1.14) 42.5 (36.5 - 49.5) p = 0.96 | 44.43 (± 2.09) 45.5 (21.5 - 55.5) p = 0.18 | 42.58 (± 1.92) 41.25 (31.5 - 58) |
| ***H19*** | 58.92 (± 2.83) 58.33 (42 - 85.67) p = 0.94 | 52.38 (± 3.18) 53 (31.33 - 69.67) p = 0.31 | 60.08 (± 4.59) 59.17 (34.33 - 89) |
| ***Dazl*** | 91.67 (± 1.07) 93.33 (84.33 - 95) p = 0.11 | 93 (± 1.74) 95 (69.33 - 98) p = 0.40 | 93.94 (± 0.44) 94.5 (91 - 95.67) |
| ***Oct4*** | 72.69 (± 1.92) 71 (63.5 - 86) p = 0.29 | 66.64 (± 1.86) 68.5 (47.5 - 74.5) p = 0.74 | 67.5 (± 4.12) 67.5 (30 - 90.5) |
| ***Abt1*** | 92.4 (± 0.80) 93 (86.5 - 96.25) p = 0.70 | 92.85 (± 0.99) 94 (81 - 97.25) p = 0.88 | 93.21 (± 0.52) 93.25 (90.25 - 95.75) |
| ***Tcf3*** | 86.67 (± 0.99) 87.25 (79.75 - 91.75) p = 0.44 | 84.27 (± 1.61) 87.5 (71 - 91) p = 0.98 | 85.54 (± 1.24) 86.13 (74.5 - 91.5) |
| **Sperm** |  |  |  |
| ***IAPs*** | 89.77 (± 0.46) 90.5 (86.5 - 91.75) p = 0.78 | 89.75 (± 0.31) 90 (87.5 - 91.5) p = 0.72 | 89.98 (± 0.60) 89.25 (87.25 - 94) |
| ***Mest*** | 21.73 (± 1.58) 22 (12.5 - 30) p = 0.59 | 21.3 (± 1.18) 21.5 (14 - 30) p = 0.53 | 22.04 (± 2.56) 19.75 (12 - 42) |
| ***Lit1*** | 31.9 (± 2.89) 33.67 (16.67 - 55) p = 0.513 | 28.76 (± 1.985) 28 (17.33 - 45.67) p = 0.9029 | 29.83 (± 3.14) 28.83 (15 - 53.33) |
| ***Snrpn*** | 19.85 (± 1.45) 20.5 (13 - 30.5) p = 0.37 | 18.93 (± 1.04) 19.5 (12 - 26.5) p = 0.31 | 18.5 (± 2.04) 17 (11 - 35) |
| ***H19*** | 82.28 (± 1.18) 83 (75.67 - 89.67) p = 0.45 | 82.51 (± 0.85) 83.33 (76.67 - 89) p = 0.16 | 83.69 (± 1.34) 85.33 (74.33 - 89.33) |
| ***Dazl*** | 34.49 (± 2.95) 36.67 (19.67 - 52.67) p = 0.48 | 29.73 (± 1.75) 31 (18.33 - 39.33) p = 0.53 | 31.64 (± 3.98) 27.33 (16.67 - 65) |
| ***Oct4*** | 39.46 (± 3.46) 39 (22 - 66) p = 0.47 | 37.83 (± 2.583) 38 (25 - 59.5) p = 0.53 | 37.82 (± 5.06) 32 (23.5 - 82) |
| ***Abt1*** | 82.38 (± 0.78) 82.75 (77.75 - 86.5) p < 0.0001 | 85.82 (± 1.44) 84.5 (78.5 - 96.5) p = 0.0024 | 93.67 (± 1.17) 95.75 (85.5 - 97) |
| ***Tcf3*** | 68.88 (± 0.85) 69 (64.75 - 75.75) p = 0.16 | 70.83 (± 1.04) 70.25 (64.25 - 79.25) p = 0.94 | 66.73 (± 4.13) 70.5 (22 - 76.5) |
