# Supplementary material for: Direct but No Transgenerational Effects of Decitabine and Vorinostat on Male Fertility
Source: PLoS One. 2015 Feb 18;10(2):e0117839. doi: 10.1371/journal.pone.0117839 (PMC4334483; doi:10.1371/journal.pone.0117839)
Supplement: S5 Table — (DOC) [file pone.0117839.s010.doc]

**Table S5:** **Body weights, data of reproductive organs and sperm parameters of DMSO treated control group and subsequent generations.** Data are shown as mean (± SEM) and median (with range). Statistical differences were calculated for the F1-, F2- and F3-DMSO control group in comparison to DMSO treated control group of the P-generation (shown as p-value). Significant nonspecific effects on subsequent generations are marked in grey.

|  | **P - DMSO control**  **(n = 16)** | **F1 - DMSO control**  **(n = 12)** | **F2 - DMSO-control**  **(n = 29)** | **F3 - DMSO control**  **(n = 31)** |
| --- | --- | --- | --- | --- |
| **Body weight [g]** | 24.88 (± 0.35) 24.5 (23 - 29) | 29.25 (± 0.77) 29 (26 - 35) p < 0.0001 | 29.72 (± 0.44) 30.1 (22.7 - 32.9) p < 0.0001 | 29 (± 0.40) 29 (24 - 34) p < 0.0001 |
| **Testes weight [mg]** | 184.4 (± 5.94) 183.6 (125 - 211) | 181.2 (± 9.09) 198.3 (123.2 - 208) p = 0.84 | 174.2 (± 6.72) 184.1 (118.3 - 215.6) p = 0.43 | 194.9 (± 4.5) 201.9 (135.5 - 229.6) p = 0.10 |
| **Testes weight/body weight [mg/g]** | 7.46 (± 0.29) 7.51 (4.31 - 8.79) | 6.147 (± 0.30) 6.33 (4.56 - 7.69) p = 0.0046 | 6.03 (± 0.19) 6.41 (4.21 - 7.43) p < 0.0001 | 6.73 (± 0.15) 7 (4.84 - 7.82) p = 0.0144 |
| **ASG weight [mg]** | 210.2 (± 10.35) 218.3 (119.4 - 283) | 189.5 (± 18.5) 193.8 (51.9 - 270.4) p = 0.47 | 218 (± 9.04) 215.3 (143.5 - 320.7) p = 0.92 | 214.3 (± 7.67) 209.9 (138.5 - 295.6) p = 0.99 |
| **Epididymides weight [mg]** | 86.61 (± 2.74) 85.75 (71 - 114.1) | 94.96 (± 4.99) 92.6 (67.6 - 127.5) p = 0.14 | 104.9 (± 2.17) 102.3 (83.2 - 128) p < 0.0001 | 114.9 (± 2.57) 117 (83.7 - 149.1) p < 0.0001 |
| **Diameter of sem.tubules [µm]** | 183 (± 6.56) 183.9 (131 - 224.4) | 174.1 (± 4.21) 177 (146.8 - 192.9) p = 0.30 | 162.8 (± 3.24) 165.1 (134.9 - 187.3) p = 0.0059 | 178.8 (± 2.54) 179.9 (155.3 - 194.3) p = 0.53 |
| **Height of sem.epithelium [µm]** | 51.49 (± 1.85) 51.93 (34.7 - 62.52) | 48.96 (± 1.59) 47.9 (40.13 - 58.32) p = 0.20 | 48.78 (± 1.17) 48.26 (38.07 - 59.52) p = 0.10 | 55.42 (± 1.24) 54.65 (44.08 - 66.52) p = 0.14 |
| **Diameter of sem.lumen [µm]** | 80.02 (± 3.7) 82.95 (53.81 - 109.9) | 76.17 (± 2.57) 74.75 (65.99 - 97.83) p = 0.56 | 65.23 (± 1.85) 64.67 (50.7 - 84.69) p = 0.0021 | 68.01 (± 1.86) 67.83 (55.18 - 83.86) p = 0.0149 |
| **Composition of testes: HC [%]** | 7.84 (± 0.39) 7.5 (4 - 13) | 8.75 (± 0.23) 9 (7 - 11) p = 0.08 | 10.75 (± 0.68) 11 (6 - 18) p = 0.0007 | 8.65 (± 0.6) 9 (4 - 15) p = 0.30 |
| **Composition of testes: 1C [%]** | 62.78 (± 0.79) 63.5 (49 - 69) | 56 (± 1.29) 59 (45 - 61) p < 0.0001 | 60.45 (± 0.78) 60 (54 - 68) p = 0.0065 | 62.25 (± 0.58) 62 (59 - 69) p = 0.06 |
| **Composition of testes: 2C [%]** | 10.38 (± 0.37)  10 (8 - 16) | 12 (± 0.48) 11 (9 - 16) p = 0.0041 | 10.5 (± 0.20) 10 (9 - 12) p = 0.29 | 10.4 (± 0.23) 10.5 (9 - 12) p = 0.42 |
| **Composition of testes: 4C [%]** | 19.09 (± 0.55) 19 (14 - 28) | 23 (± 0.85) 22 (18 - 31) p = 0.0002 | 18.4 (± 0.41) 19 (15 - 23) p = 0.43 | 18.4 (± 0.38) 19 (15 - 21) p = 0.5 |
| **Efficiency of spermatogenesis [%]** | 0.96 (± 0.01) 0.96 (0.92 - 1) | 0.98 (± 0.01) 0.98 (0.94 - 1) p = 0.27 | 0.98 (± 0.01) 0.98 (0.94 - 1) p = 0.12 | 0.96 (± 0.01) 0.96 (0.9 - 1) p = 0.58 |
| **Sperm concentration [mill/ml]** | 29.33 (± 2.99) 27.25 (14.25 - 57) | 34.52 (± 3.23) 36.98 (13.36 - 47.31) p = 0.25 | 40.43 (± 2.73) 41.25 (11.1 - 67.75) p = 0.0132 | 36.38 (± 1.7) 36.38 (20 - 52.5) p = 0.0317 |
| **Progressive sperm motility [%]** | 52.72 (± 1.3) 54 (44.5 - 60.75) | 62.73 (± 1.59) 62.13 (52.75 - 71) p = 0.0003 | 59.65 (± 1.40) 60.75 (35.75 - 75.5) p = 0.0008 | 70.04 (± 0.81) 71.25 (60.25 - 76.5) p < 0.0001 |
| **Sperm vitality [%]** | 70.48 (± 1.58) 71.13 (58.25 - 78) | 68.33 (± 1.96) 71.38 (53.25 - 74.25) p = 0.4 | 47.56 (± 1.49) 47.75 (34 - 68) p < 0.0001 | 49.19 (± 0.85) 49 (40 - 59.5) p < 0.0001 |
| **Normal sperm morphology [%]** | 58.5 (± 2.21) 59 (42.5 - 70) | 65.42 (± 2.04) 65.75 (53 - 77) p = 0.0484 | 61.88 (± 2.37) 62.5 (41.5 - 77.5) p = 0.48 | 66.33 (± 1.47) 66.25 (48 - 75) p = 0.0082 |
| **DNA fragmentation [%]** | 3.216 (± 0.96) 2.3 (1.06 - 17.48) | 7.8 (± 1.27) 6.67 (2.83 - 14.46) p = 0.0002 | 6.45 (± 1.74) 4.17 (2.08 - 35.75) p = 0.0007 | 4.38 (± 0.41) 4.07 (2.02 - 9.83) p = 0.0001 |
