# Supplementary material for: Direct but No Transgenerational Effects of Decitabine and Vorinostat on Male Fertility
Source: PLoS One. 2015 Feb 18;10(2):e0117839. doi: 10.1371/journal.pone.0117839 (PMC4334483; doi:10.1371/journal.pone.0117839)
Supplement: S6 Table — (DOC) [file pone.0117839.s011.doc]

**Table S6:** **DNA methylation of blood and spermatozoa of the DMSO treated control group and subsequent generations.** Data are shown as mean [%] (± SEM) and median [%] (with range). Statistical differences were calculated for the F1-, F2- and F3-DMSO control group in comparison to DMSO treated control group of the P-generation (shown as p-value and marked in grey).

|  | **P - DMSO control**  **(n = 16)** | **F1 - DMSO control**  **(n = 12)** | **F2 - DMSO-control**  **(n = 20)** | **F3 - DMSO control**  **(n =20)** |
| --- | --- | --- | --- | --- |
| **Blood** |  |  |  |  |
| ***IAPs*** | 95.42 (± 0.21) 95.75 (93.5 - 96.25) | 95.02 (± 0.13) 95 (94.25 - 96) p = 0.09 | 94.7 (± 0.15) 94.63 (93 - 95.75) p = 0.0048 | 94.2 (± 0.21) 94.5 (92.25 - 95.5) p = 0.0006 |
| ***Mest*** | 47.07 (± 0.51) 47 (44 - 50.5) | 45.33 (± 0.7) 45.75 (41 - 49) p = 0.1 | 46.55 (± 0.61) 46.75 (38 - 51) p = 0.85 | 48.45 (± 0.7) 48 (43.5 - 54.5) p = 0.22 |
| ***Lit1*** | 57.24 (± 1.07) 57.67 (50 - 67.33) | 56.94 (± 1.14) 58.17 (47.67 - 62.67) p = 0.9 | 57.38 (± 0.7) 56.67 (52.33 - 66.67) p = 0.87 | 53.85 (± 1) 53.17 (46.33 - 62.33) p = 0.0301 |
| ***Snrpn*** | 41.67 (± 0.71) 41 (37.5 - 47.5) | 42.58 (± 1.92) 41.25 (31.5 - 58) p = 0.92 | 40.68 (± 0.74) 41.5 (33 - 46) p = 0.65 | 39.73 (± 1.16) 40.5 (26 - 48) p = 0.34 |
| ***H19*** | 59.98 (± 1.20) 59 (52.33 - 68.33) | 60.08 (± 4.59) 59.17 (34.33 - 89) p = 0.83 | 58.53 (± 1.58) 59.17 (42.67 - 71.33) p = 0.84 | 62.22 (± 2.69) 62.17 (34.33 - 89) p = 0.38 |
| ***Dazl*** | 95.02 (± 0.2) 95.33 (93.67 - 96.33) | 93.94 (± 0.44) 94.5 (91 - 95.67) p = 0.07 | 95.67 (± 0.23) 96 (93.67 - 97) p = 0.0267 | 92.9 (± 0.9) 94.17 (80.33 - 98.33) p = 0.0065 |
| ***Oct4*** | 68.97 (± 0.82) 68 (63.5 - 73.5) | 67.5 (± 4.12) 67.5 (30 - 90.5) p = 0.81 | 71.06 (± 1.04) 70.75 (65 - 84.5) p = 0.29 | 67.6 (± 0.8) 68 (58 - 73) p = 0.29 |
| ***Abt1*** | 97.05 (± 0.21) 97.25 (94.75 – 97.75) | 93.21 (± 0.52) 93.25 (90.25 - 95.75) p < 0.0001 | 95.86 (± 0.09) 95.88 (95 - 96.5) p < 0.0001 | 95.18 (± 0.61) 95.88 (85.25 - 97.75) p = 0.0003 |
| ***Tcf3*** | 90.93 (± 0.70) 90.75 (83 - 95.25) | 85.54 (± 1.24) 86.13 (74.5 - 91.5) p = 0.0005 | 87.56 (± 1.14) 88.88 (71 - 92) p = 0.0084 | 82.24 (± 1.10) 83.25 (69.25 - 88) p < 0.0001 |
| **Sperm** |  |  |  |  |
| ***IAPs*** | 87.48 (± 0.34) 87.5 (86 – 91) | 89.98 (± 0.6) 89.25 (87.25 - 94) p = 0.0007 | 89.65 (± 0.4) 90.25 (86 - 92.25) p = 0.0003 | 89.41 (± 0.32) 89.5 (86.75 - 92) p = 0.0002 |
| ***Mest*** | 14.97 (± 1.05) 14.25 (10.5 - 25) | 22.04 (± 2.56) 19.75 (12 - 42) p = 0.0081 | 21.23 (± 1.36) 22 (12.5 - 33) p = 0.0014 | 24.08 (± 1.38) 21.75 (14.5 - 37.5) p < 0.0001 |
| ***Lit1*** | 21.94 (± 1.6) 20.17 (13.33 - 36.67) | 29.83 (± 3.14) 28.83 (15 - 53.33) p = 0.0308 | 29.08 (± 1.62) 27 (20 - 43) p = 0.002 | 29.07 (± 1.38) 27.67 (17.33 - 38.67) p = 0.0019 |
| ***Snrpn*** | 11.91 (± 0.93) 10.75 (7 - 19.5) | 18.5 (± 2.04) 17 (11 - 35) p = 0.0043 | 18.75 (± 1.21) 18 (11.5 - 30.5) p = 0.0002 | 18.75 (± 1.09) 18.25 (10 - 28) p = 0.0002 |
| ***H19*** | 86.73 (± 0.61) 87 (83 - 91) | 83.69 (± 1.34) 85.33 (74.33 - 89.33) p = 0.12 | 81.27 (± 1.06) 82.17 (73 - 90) p = 0.0004 | 82.65 (± 1) 83 (73.67 - 91) p = 0.0029 |
| ***Dazl*** | 25.25 (± 1.57) 23.83 (18.33 – 38.67) | 31.64 (± 3.98) 27.33 (16.67 - 65) p = 0.15 | 31.05 (± 1.97) 31 (17 - 47.33) p = 0.05 | 32.45 (± 1.51) 32.67 (19.67 - 44.67) p = 0.0046 |
| ***Oct4*** | 31.88 (± 1.85) 30.75 (19 – 49) | 37.82 (± 5.06) 32 (23.5 - 82) p = 0.51 | 43.28 (± 2.83) 43 (23 - 68) p = 0.0053 | 42.63 (± 2.19) 44.5 (23.5 - 57.5) p = 0.0009 |
| ***Abt1*** | 79.16 (± 0.56) 78.88 (76.25 - 84.5) | 93.67 (± 1.17) 95.75 (85.5 - 97) p < 0.0001 | 85.13 (± 0.6) 85.25 (80.75 - 90) p < 0.0001 | 89.09 (± 0.99) 88.13 (83 - 96.75) p < 0.0001 |
| ***Tcf3*** | 69.55 (± 0.49) 69.25 (65.75 – 73.75) | 66.73 (± 4.13) 70.5 (22 - 76.5) p = 0.39 | 70.98 (± 0.67) 70.38 (66.75 - 81) p = 0.1 | 64.91 (± 0.65) 65.13 (58.75 - 68.75) p < 0.0001 |
