# Supplementary material for: Direct but No Transgenerational Effects of Decitabine and Vorinostat on Male Fertility
Source: PLoS One. 2015 Feb 18;10(2):e0117839. doi: 10.1371/journal.pone.0117839 (PMC4334483; doi:10.1371/journal.pone.0117839)
Supplement: S7 Table — (DOC) [file pone.0117839.s012.doc]

**Table S7**: **Body weights, data of reproductive organs and sperm parameters of the F2- and F3-generations.** Data are shown as mean (± SEM) and median (with range). Statistical differences were calculated for decitabine and vorinostat in comparison to DMSO vehicle control (shown as p-value). Significant transgenerational effects of decitabine and vorinostat are marked in grey.

|  | **F2-generation** | | |
| --- | --- | --- | --- |
|  | **Decitabine**  **(n = 32)** | **Vorinostat**  **(n = 22)** | **DMSO-control**  **(n = 29)** |
| **Body weight [g]** | 29.38 (± 0.34) 29.15 (25.9 - 33.5) p = 0.26 | 30.4 (± 0.51) 31.05 (24.7 - 33.4) p = 0.18 | 29.72 (± 0.44) 30.1 (22.7 – 32.9) |
| **Testes weight [mg]** | 179.8 (± 5.08) 191.6 (117.9 - 218.8) p = 0.77 | 195.6 (± 4.82) 201.2 (145 - 222.9) p = 0.0294 | 178.8 (± 5.71) 185.6 (118.3 - 232.5) |
| **Testes weight/body weight [mg/g]** | 6.05 (± 0.23) 6.63 (2.64 - 7.99) p = 0.54 | 6.44 (± 0.13) 6.49 (5.31 - 7.41) p = 0.34 | 6.03 (± 0.19) 6.41 (4.21 - 7.43) |
| **ASG weight [mg]** | 202.3 (± 4.96) 205.9 (151.1 - 258.9) p = 0.27 | 204.6 (± 10.79) 209.1 (121.7 - 320) p = 0.39 | 218 (± 9.04) 215.3 (143.5 - 320.7) |
| **Epididymides weight [mg]** | 100.9 (± 1.99) 100.7 (76.6 - 123.7) p = 0.23 | 109.9 (± 2.97) 107.9 (87.5 - 130.8) p = 0.24 | 104.9 (± 2.17) 102.3 (83.2 - 128) |
| **Diameter of sem.tubules [µm]** | 164.9 (± 3.72) 162.2 (132.5 - 195.2) p = 0.67 | 173.3 (± 3.39) 171.9 (132.6 - 193.5) p = 0.0305 | 162.8 (± 3.24) 165.1 (134.9 - 187.3) |
| **Height of sem.epithelium [µm]** | 47.84 (± 1.23) 48.83 (37.94 - 55.31) p = 0.76 | 52.14 (± 1.12) 53.85 (41.62 - 59.5) p = 0.0315 | 48.78 (± 1.17) 48.26 (38.07 – 59.52) |
| **Diameter of sem.lumen [µm]** | 69.25 (± 2.40) 67.06 (50.12 - 96.26) p = 0.21 | 69.04 (± 2.18) 66.59 (49.4 - 83.21) p = 0.15 | 65.23 (± 1.85) 64.67 (50.7 – 84.69) |
| **Composition of testes: HC [%]** | 9.9 (± 0.62) 9 (7 - 18) p = 0.3 | 10.5 (± 0.65) 10 (6 - 15) p = 0.83 | 10.75 (± 0.68) 11 (6 - 18) |
| **Composition of testes: 1C [%]** | 56.4 (± 1.47) 58 (45 - 67) p = 0.06 | 58.1 (± 0.98) 57.5 (51 - 65) p = 0.1 | 60.45 (± 0.78) 60 (54 - 68) |
| **Composition of testes: 2C [%]** | 12 (± 0.46) 12 (9 - 16) p = 0.0145 | 11.3 (± 0.36) 11 (9 - 14) p = 0.13 | 10.5 (± 0.2) 10 (9 - 12) |
| **Composition of testes: 4C [%]** | 21.85 (± 1.03) 20 (15 - 30) p = 0.0066 | 20.1 (± 0.74) 20 (14 - 26) p = 0.11 | 18.4 (± 0.41) 19 (15 - 23) |
| **Efficiency of spermatogenesis [%]** | 0.97 (± 0.01) 0.98 (0.94 - 1) p = 0.48 | 0.98 (± 0.01) 0.98 (0.94 - 1) p = 0.65 | 0.98 (± 0.01) 0.98 (0.94 - 1) |
| **Sperm concentration [mill/ml]** | 38.14 (± 2.21) 38.98 (12 - 72.5) p = 0.51 | 46.7 (± 3.71) 47.25 (7.3 - 79.5) p = 0.17 | 40.43 (± 2.73) 41.25 (11.1 – 67.75) |
| **Progressive sperm motility [%]** | 59.63 (± 1.48) 60.63 (31.5 - 72) p = 0.79 | 55.56 (± 1.93) 57.38 (27 - 70.5) p = 0.11 | 59.65 (± 1.4) 60.75 (35.75 - 75.5) |
| **Sperm vitality [%]** | 46.72 (± 1.2) 45.13 (28.5 - 63.25) p = 0.66 | 46.13 (± 1.46) 45.75 (32.5 - 60) p = 0.5 | 47.56 (± 1.49) 47.75 (34 - 68) |
| **Normal sperm morphology [%]** | 62.35 (± 1.61) 61.25 (52.5 - 79.5) p = 1 | 64.43 (± 1.48) 65.25 (53.5 - 78) p = 0.43 | 61.88 (± 2.37) 62.5 (41.5 – 77.5) |
| **DNA fragmentation [%]** | 5.44 (± 1.1) 4.08 (2.04 - 24.32) p = 0.77 | 7.92 (± 2.29) 4.41 (2.49 - 46.65) p = 0.47 | 6.45 (± 1.74) 4.17 (2.08 - 35.75) |
|  | **F3-generation** | | |
|  | **Decitabine**  **(n = 33)** | **Vorinostat**  **(n = 41)** | **DMSO-control**  **(n = 31)** |
| **Body weight [g]** | 30 (± 0.31) 30 (27 - 34) p = 0.06 | 29.07 (± 0.4) 29 (22 - 33) p = 0.68 | 29 (± 0.40) 29 (24 – 34) |
| **Testes weight [mg]** | 186.5 (± 5.77) 196.8 (111.8 - 232.9) p = 0.33 | 193.9 (± 3.92) 201.2 (125.9 - 240.7) p = 0.74 | 194.9 (± 4.5) 201.9 (135.5 - 229.6) |
| **Testes weight/body weight [mg/g]** | 6.23 (± 0.2) 6.3 (3.73 - 8.03) p = 0.08 | 6.71 (± 0.13) 6.94 (4.97 - 7.97) p = 0.81 | 6.73 (± 0.15) 7 (4.84 - 7.82) |
| **ASG weight [mg]** | 219.9 (± 6.26) 219.9 (143.1 - 286.8) p = 0.37 | 211.8 (± 8.03) 219.2 (100.7 - 356.3) p = 0.89 | 214.3 (± 7.67) 209.9 (138.5 - 295.6) |
| **Epididymides weight [mg]** | 111.3 (± 2.75) 110.5 (81.6 - 153) p = 0.19 | 115.5 (± 2.32) 113.6 (81.5 - 146.1) p = 0.9 | 114.9 (± 2.57) 117 (83.7 – 149.1) |
| **Diameter of sem.tubules [µm]** | 176.9 (± 2.93) 179.2 (150.4 - 199.1) p = 0.61 | 177.9 (± 2.55) 178.2 (158.6 - 204.8) p = 0.8 | 178.8 (± 2.54) 179.9 (155.3 - 194.3) |
| **Height of sem.epithelium [µm]** | 55.97 (± 2.47) 56.45 (28.75 - 90.26) p = 0.66 | 55.9 (± 0.96) 56.17 (47.66 - 62) p = 0.68 | 55.42 (± 1.24) 54.65 (44.08 – 66.52) |
| **Diameter of sem.lumen [µm]** | 66.84 (± 1.7) 68.55 (52.05 - 78.66) p = 0.72 | 66.11 (± 1.69) 66.13 (55.17 - 82.1) p = 0.39 | 68.01 (± 1.86) 67.83 (55.18 – 83.86) |
| **Composition of testes: HC [%]** | 8.45 (± 0.48) 8.5 (5 - 12) p = 0.99 | 8.55 (± 0.55) 9 (4 - 13) p = 0.97 | 8.65 (± 0.6) 9 (4 - 15) |
| **Composition of testes: 1C [%]** | 62.5 (± 0.6) 62.5 (56 - 70) p = 0.43 | 61.2 (± 1.11) 62 (45 - 66) p = 0.97 | 62.25 (± 0.58) 62 (59 - 69) |
| **Composition of testes: 2C [%]** | 10.7 (± 0.25) 11 (8 - 13) p = 0.39 | 10.9 (± 0.33) 10 (9 - 15) p = 0.46 | 10.4 (± 0.23) 10.5 (9 – 12) |
| **Composition of testes: 4C [%]** | 18.3 (± 0.36) 18 (15 - 22) p = 0.77 | 19.15 (± 0.67) 18 (16 - 28) p = 0.73 | 18.4 (± 0.38) 19 (15 - 21) |
| **Efficiency of spermatogenesis [%]** | 0.97 (± 0.01) 0.97 (0.92 - 1) p = 0.24 | 0.96 (± 0.01) 0.97 (0.92 - 1) p = 0.53 | 0.96 (± 0.01) 0.96 (0.9 - 1) |
| **Sperm concentration [mill/ml]** | 35.57 (± 2.27) 34.5 (14 - 56.25) p = 0.78 | 37.12 (± 2.42) 31 (13.1 - 70.5) p = 0.82 | 36.38 (± 1.7) 36.38 (20 – 52.5) |
| **Progressive sperm motility [%]** | 69.9 (± 0.8) 70.25 (55.75 - 78.75) p = 0.86 | 70.71 (± 0.66) 71.25 (61.5 - 80.75) p = 0.75 | 70.04 (± 0.81) 71.25 (60.25 - 76.5) |
| **Sperm vitality [%]** | 44.73 (± 1) 45.25 (28.75 - 60) p = 0.0013 | 43.47 (± 0.8) 43.25 (32.5 - 57.25) p < 0.0001 | 49.19 (± 0.85) 49 (40 - 59.5) |
| **Normal sperm morphology [%]** | 66.3 (± 1.44) 67.25 (48.5 - 74) p = 0.79 | 63.63 (± 1.8) 64.25 (49.5 - 79) p = 0.26 | 66.33 (± 1.47) 66.25 (48 - 75) |
| **DNA fragmentation [%]** | 4.09 (± 0.42) 3.49 (1.76 - 7.99) p = 0.51 | 5.14 (± 0.67) 4.43 (2.09 - 15.25) p = 0.51 | 4.38 (± 0.41) 4.07 (2.02 - 9.83) |
