# Supplementary material for: Direct but No Transgenerational Effects of Decitabine and Vorinostat on Male Fertility
Source: PLoS One. 2015 Feb 18;10(2):e0117839. doi: 10.1371/journal.pone.0117839 (PMC4334483; doi:10.1371/journal.pone.0117839)
Supplement: S8 Table — (DOC) [file pone.0117839.s013.doc]

**Table S8**: **DNA methylation of blood and spermatozoa of the F2- and F3-generations.** Data are shown as mean [%] (± SEM) and median [%] (with range). Statistical differences were calculated for decitabine and vorinostat in comparison to DMSO vehicle control (shown as p-value). Significant transgenerational effects of decitabine and vorinostat are marked in grey.

| **F2-generation** | | | |
| --- | --- | --- | --- |
|  | **Decitabine**  **(n = 20)** | **Vorinostat**  **(n = 20)** | **DMSO-control**  **(n = 20)** |
| **Blood** |  |  |  |
| ***IAPs*** | 94.64 (± 0.18) 94.75 (92.75 - 95.75) p = 0.9 | 94.33 (± 0.27) 94.75 (91.25 - 95.5) p = 0.51 | 94.7 (± 0.15) 94.63 (93 - 95.75) |
| ***Mest*** | 48.1 (± 0.57) 47.75 (43 - 53) p = 0.12 | 49.18 (± 0.67) 48.5 (44.5 - 56.5) p = 0.01 | 46.55 (± 0.61) 46.75 (38 - 51) |
| ***Lit1*** | 57.37 (± 0.55) 57.83 (51.33 - 62) p = 0.6 | 57.37 (± 1.64) 59.17 (28 - 63) p = 0.16 | 57.38 (± 0.7) 56.67 (52.33 - 66.67) |
| ***Snrpn*** | 41.63 (± 0.94) 42 (32 - 51.5) p = 0.54 | 44 (± 2.62) 42.25 (34 - 90.5) p = 0.46 | 40.68 (± 0.74) 41.5 (33 – 46) |
| ***H19*** | 61.02 (± 1.3) 61.33 (53 - 74) p = 0.51 | 57.75 (± 2.45) 58.83 (25.33 - 79.67) p = 0.77 | 58.53 (± 1.58) 59.17 (42.67 – 71.33) |
| ***Dazl*** | 95.73 (± 0.18) 95.67 (93.33 - 97) p = 0.87 | 95.63 (± 0.28) 95.33 (94 - 99.33) p = 0.67 | 95.67 (± 0.23) 96 (93.67 - 97) |
| ***Oct4*** | 72.15 (± 1.19) 71.75 (63 - 82.5) p = 0.49 | 70.56 (± 1.31) 71 (59 - 81) p = 0.96 | 71.06 (± 1.04) 70.75 (65 - 84.5) |
| ***Abt1*** | 94.61 (± 1.67) 96.5 (63 - 97.5) p = 0.0089 | 95.83 (± 0.43) 96.5 (89 - 97.5) p = 0.0308 | 95.86 (± 0.09) 95.88 (95 - 96.5) |
| ***Tcf3*** | 86.91 (± 1.04) 88.5 (76.5 - 92.75) p = 0.5 | 86.98 (± 0.98) 87.5 (74.5 - 97) p = 0.16 | 87.56 (± 1.14) 88.88 (71 - 92) |
| **Sperm** |  |  |  |
| ***IAPs*** | 88.56 (± 0.42) 88.75 (85.25 - 92) p = 0.07 | 89.31 (± 0.43) 89.5 (85.75 - 92.75) p = 0.57 | 89.65 (± 0.4) 90.25 (86 - 92.25) |
| ***Mest*** | 26.75 (± 1.5) 25.75 (14 - 45.5) p = 0.0093 | 22.48 (± 1.53) 21.25 (12 - 34.5) p = 0.62 | 21.23 (± 1.36) 22 (12.5 – 33) |
| ***Lit1*** | 28.3 (± 1.57) 28.67 (15.67 - 47) p = 0.8 | 29.7 (± 2.07) 31.5 (14.33 - 44.67) p = 0.72 | 29.08 (± 1.62) 27 (20 - 43) |
| ***Snrpn*** | 17.55 (± 1.18) 17.75 (10 - 29.5) p = 0.45 | 19.38 (± 1.43) 18 (9 - 30) p = 0.74 | 18.75 (± 1.21) 18 (11.5 - 30.5) |
| ***H19*** | 81.87 (± 1.42) 82.17 (65 - 91.67) p = 0.54 | 82.05 (± 0.99) 83.17 (73.67 - 89.67) p = 0.64 | 81.27 (± 1.06) 82.17 (73 - 90) |
| ***Dazl*** | 28.43 (± 2.02) 29.5 (14.33 - 47.67) p = 0.34 | 32.37 (± 2.45) 30.83 (15.33 - 53.33) p = 0.79 | 31.05 (± 1.97) 31 (17 - 47.33) |
| ***Oct4*** | 39.03 (± 2.66) 43 (22 - 57) p = 0.31 | 44.58 (± 3.18) 44 (23.5 - 69.5) p = 0.72 | 43.28 (± 2.83) 43 (23 - 68) |
| ***Abt1*** | 84.85 (± 0.68) 84.5 (80.75 - 93.75) p = 0.54 | 86.51 (± 0.87) 86.38 (81 - 96.75) p = 0.27 | 85.13 (± 0.6) 85.25 (80.75 - 90) |
| ***Tcf3*** | 71.61 (± 0.48) 71.38 (67.25 - 75) p = 0.14 | 70.66 (± 0.57) 70.25 (66.25 - 77.25) p = 0.81 | 70.98 (± 0.67) 70.38 (66.75 - 81) |
| **F3-generation** | | | |
|  | **Decitabine**  **(n = 20)** | **Vorinostat**  **(n = 20)** | **DMSO-control**  **(n = 20)** |
| **Blood** |  |  |  |
| ***IAPs*** | 94.46 (± 0.19) 94.5 (92.5 - 96) p = 0.43 | 94.19 (± 0.26) 94.25 (90.25 - 95.5) p = 0.94 | 94.2 (± 0.21) 94.5 (92.25 - 95.5) |
| ***Mest*** | 49.03 (± 0.63) 49.5 (41.5 - 54) p = 0.34 | 49.03 (± 0.89) 49.5 (37 - 54.5) p = 0.34 | 48.45 (± 0.7) 48 (43.5 - 54.5) |
| ***Lit1*** | 53.38 (± 0.74) 54 (42.67 - 58) p = 0.97 | 53.72 (± 0.76) 53.83 (47.33 - 59.67) p = 0.95 | 53.85 (± 1) 53.17 (46.33 - 62.33) |
| ***Snrpn*** | 42.43 (± 1.25) 43 (27 - 53) p = 0.045 | 40.8 (± 1.08) 41.25 (26.5 - 52.5) p = 0.64 | 39.73 (± 1.16) 40.5 (26 – 48) |
| ***H19*** | 59.82 (± 1.65) 58.83 (49 - 74.33) p = 0.27 | 58.6 (± 1.36) 60 (43.33 - 71.33) p = 0.17 | 62.22 (± 2.69) 62.17 (34.33 - 89) |
| ***Dazl*** | 93.27 (± 0.57) 93.67 (84.33 - 96) p = 0.78 | 93.65 (± 0.38) 94.33 (89 - 95.67) p = 0.89 | 92.9 (± 0.9) 94.17 (80.33 - 98.33) |
| ***Oct4*** | 68.32 (± 1.4) 68.5 (53.5 - 76.5) p = 0.48 | 67.3 (± 0.77) 67.25 (60.5 - 74.5) p = 0.51 | 67.6 (± 0.8) 68 (58 – 73) |
| ***Abt1*** | 95.56 (± 0.36) 96.5 (91.75 - 97.5) p = 0.94 | 95.88 (± 0.39) 96.5 (91 - 97.5) p = 0.22 | 95.18 (± 0.61) 95.88 (85.25 – 97.75) |
| ***Tcf3*** | 76.48 (± 2.27) 81.13 (50.5 - 86.75) p = 0.0219 | 80.58 (± 1.32) 82.13 (64 - 87.75) p = 0.27 | 82.24 (± 1.1) 83.25 (69.25 - 88) |
| **Sperm** |  |  |  |
| ***IAPs*** | 88.53 (± 0.39) 88.75 (84.75 - 91) p = 0.08 | 88.59 (± 0.38) 89 (84.25 - 90.5) p = 0.11 | 89.41 (± 0.32) 89.5 (86.75 - 92) |
| ***Mest*** | 22.9 (± 1.56) 22.5 (11.5 - 37.5) p = 0.61 | 22.2 (± 1.67) 23.25 (5.5 - 34.5) p = 0.47 | 24.08 (± 1.38) 21.75 (14.5 - 37.5) |
| ***Lit1*** | 27.98 (± 2.47) 25 (14 - 59) p = 0.39 | 26.17 (± 1.93) 26 (5 - 40) p = 0.3 | 29.07 (± 1.38) 27.67 (17.33 – 38.67) |
| ***Snrpn*** | 17.05 (± 1.48) 16 (7 - 30) p = 0.27 | 16.55 (± 1.29) 16.25 (4 - 26) p = 0.22 | 18.75 (± 1.09) 18.25 (10 - 28) |
| ***H19*** | 83.47 (± 1.26) 84.33 (69 - 92.67) p = 0.45 | 83.9 (± 1.16) 83.67 (71.33 - 93.33) p = 0.27 | 82.65 (± 1) 83 (73.67 - 91) |
| ***Dazl*** | 29.82 (± 2.28) 29.33 (13.67 - 57.67) p = 0.21 | 29.43 (± 2.18) 30.33 (5.333 - 42.67) p = 0.38 | 32.45 (± 1.51) 32.67 (19.67 – 44.67) |
| ***Oct4*** | 34.71 (± 2.7) 31.5 (18.5 - 66.5) p = 0.011 | 34.12 (± 3.15) 32.5 (11.5 - 58) p = 0.0316 | 42.63 (± 2.19) 44.5 (23.5 – 57.5) |
| ***Abt1*** | 90.58 (± 1.02) 91.38 (80 - 96.5) p = 0.25 | 90.26 (± 0.96) 90.5 (79.75 - 96.75) p = 0.34 | 89.09 (± 0.99) 88.13 (83 - 96.75) |
| ***Tcf3*** | 65.34 (± 1.29) 66.25 (43.75 - 72.5) p = 0.34 | 66.4 (± 0.54) 66.63 (59.75 - 70.75) p = 0.08 | 64.91 (± 0.65) 65.13 (58.75 – 68.75) |
